# Impaired male fertility and abnormal epididymal epithelium differentiation in mice lacking CRISP1 and CRISP4

**DOI:** 10.1038/s41598-018-35719-3

**Published:** 2018-12-03

**Authors:** Guillermo Carvajal, Nicolás Gastón Brukman, Mariana Weigel Muñoz, María A. Battistone, Vanesa A. Guazzone, Masahito Ikawa, Miyata Haruhiko, Livia Lustig, Sylvie Breton, Patricia S. Cuasnicu

**Affiliations:** 1 Instituto de Biología y Medicina Experimental (IByME-CONICET), Buenos Aires, C1428ADN Argentina; 2 Program in Membrane Biology, Center for Systems Biology, Nephrology Division, and Department of Medicine, Massachusetts General Hospital, Harvard Medical School, Boston, Massachusetts, USA; 3 Instituto de Investigaciones Biomédicas (INBIOMED-UBA-CONICET), Buenos Aires, C1121ABG Argentina; 4 Research Institute for Microbial Diseases, Osaka University, Osaka, 565-0871 Japan

## Abstract

Epididymal Cysteine Rich Secretory Proteins 1 and 4 (CRISP1 and CRISP4) associate with sperm during maturation and play different roles in fertilization. However, males lacking each of these molecules individually are fertile, suggesting compensatory mechanisms between these homologous proteins. Based on this, in the present work, we generated double CRISP1/CRISP4 knockout (DKO) mice and examined their reproductive phenotype. Our data showed that the simultaneous lack of the two epididymal proteins results in clear fertility defects. Interestingly, whereas most of the animals exhibited specific sperm fertilizing ability defects supportive of the role of CRISP proteins in fertilization, one third of the males showed an unexpected epididymo-orchitis phenotype with altered levels of inflammatory molecules and non-viable sperm in the epididymis. Further analysis showed that DKO mice exhibited an immature epididymal epithelium and abnormal luminal pH, supporting these defects as likely responsible for the different phenotypes observed. These observations reveal that CRISP proteins are relevant for epididymal epithelium differentiation and male fertility, contributing to a better understanding of the fine-tuning mechanisms underlying sperm maturation and immunotolerance in the epididymis with clear implications for human epididymal physiology and pathology.

## Introduction

Gamete interaction in mammals involves a series of sequential and coordinated steps that culminates in the union of the sperm and egg genomes. Differently from lower vertebrates, the highly differentiated mammalian sperm that leave the testes do not have the ability to fertilize an egg. This requires that sperm first undergo a maturation process while passing through the epididymis where they will acquire progressive motility and the ability to recognize and interact with the egg^1,2^. After ejaculation, mature epididymal sperm need to undergo another process known as capacitation that occurs while ascending the female tract and which enables sperm both to undergo the acrosomal reaction, an exocytotic event that takes place in the head, and to develop a vigorous flagellar movement termed hyperactivation^3^. Although the mechanisms underlying the acquisition of sperm fertilizing ability are still under investigation^4,5^, it is known that sperm maturation involves numerous changes at the gamete surface level, most of which occur as a result of the association of epididymal proteins with the sperm plasma membrane^6,7^. Two such proteins are CRISP1 and CRISP4^8–11^ which belong to a highly conserved and widely distributed family among vertebrates known as Cysteine RIch Secretory Proteins (CRISP). In mammals, the CRISP family comprises four members (CRISP1–4) (20–30 kDa) mainly expressed in the male reproductive tract and characterized by the presence of sixteen conserved cysteines, ten of which are located in the C-terminal region or cysteine-rich domain (CRD) connected to the plant pathogenesis-related 1 (PR-1) domain located in the N-terminus^12,13^. Evidence suggests that CRISP proteins have evolved to perform a variety of functions that rely on these two different domains^13–15^.

Using different genetic, biochemical and molecular *in vivo* and *in vitro* approaches, we found that androgen-dependent epididymal CRISP1, identified by our group^8^ associates with the sperm surface during epididymal transit giving rise to two populations of the protein in the mature sperm: one loosely associated that is released from sperm during capacitation and, thus, proposed as a decapacitating factor^16–18^, and one firmly attached that remained in capacitated sperm and was reported to participate in fertilization, more specifically in sperm-ZP interaction and gamete fusion through its binding to egg complementary sites^19,20^. Novel results from our group showed that CRISP1 also has the ability to inhibit CatSper^21^, the main calcium channel present in mammalian sperm and essential for hyperactivation development^22,23^ and male fertility^24–26^. Thus, whereas the gamete fusion ability of CRISP1 was reported to reside within the PR-1 domain^15^, it is likely that the ability of CRISP1 to inhibit CatSper is located within CRD portion of the molecule known to have ion channel regulatory activity in CRISP proteins from both reptiles^27,28^ and mammals^14,29^. In spite of the fact that mutant sperm lacking CRISP1 exhibit clear sperm functional defects, CRISP1-null males are fertile^21,30,31^, opening the possibility that another homologous CRISP protein compensates for the lack of CRISP1.

The other epididymal CRISP protein is CRISP4, the last identified member of the family^10^ and less characterized than epididymal CRISP1. Evidence revealed that CRISP4 is also synthesized in response to androgens, associates with sperm during epididymal maturation and has roles in both calcium channel (i.e. TRPM8) and acrosome reaction regulation^29^, as well as in sperm binding to the ZP^32^. As observed for CRISP1, CRISP4 null males have *in vitro* fertilization defects but normal animal fertility, supporting the existence of compensatory mechanisms between the two homologous epididymal CRISP proteins. Unlike mice, humans have only three CRISP proteins and epididymal human CRISP1 (hCRISP1) is considered the homolog of both rodent CRISP1 and CRISP4 based on sequence identity and similar functions (i.e. roles in the gamete fusion and sperm-ZP binding)^33,34^.

In view of this, in the present work, we analyzed the reproductive phenotype of mice lacking both CRISP1 and CRISP4 revealing the relevance of these proteins for male fertility as well as for proper epididymal epithelium differentiation and luminal acidification.

## Results

### Generation of CRISP1/CRISP4 double knockout mice

Double CRISP1/CRISP4 knockout (DKO) mice were generated by mating animals from C57BL/6 (B6) *Crisp1*^*−/−*^ and C57BL/6*DBA (B6D2) *Crisp4*^*−/−*^ single KO available at our laboratory. Considering the influence of the genetic background on the final phenotype of mutant mice and that the B6 CRISP1 KO colony had already been characterized^31^, we decided to first analyze the phenotype of the B6D2 CRISP4 KO mice not previously reported. Results showed that these mutant males lacking CRISP4 (Fig. S1) were fertile and had sperm that fertilized normally *in vivo* (Table S1) but exhibited impaired ability to fertilize cumulus-oocyte complexes (COC), ZP-intact and ZP-free eggs *in vitro* (Fig. S2A–C). Further examination revealed that *Crisp4*^*−/−*^ sperm showed normal viability, motility and ability to penetrate the cumulus mass, bind to the ZP and develop hyperactivated motility (Table S1) but failed to undergo both protein tyrosine phosphorylation and the progesterone-induced acrosome reaction during capacitation (Fig. S2D,E).

### The lack of CRISP1 and CRISP4 affects *in vivo* fertilization and male fertility

**Figure 1:**
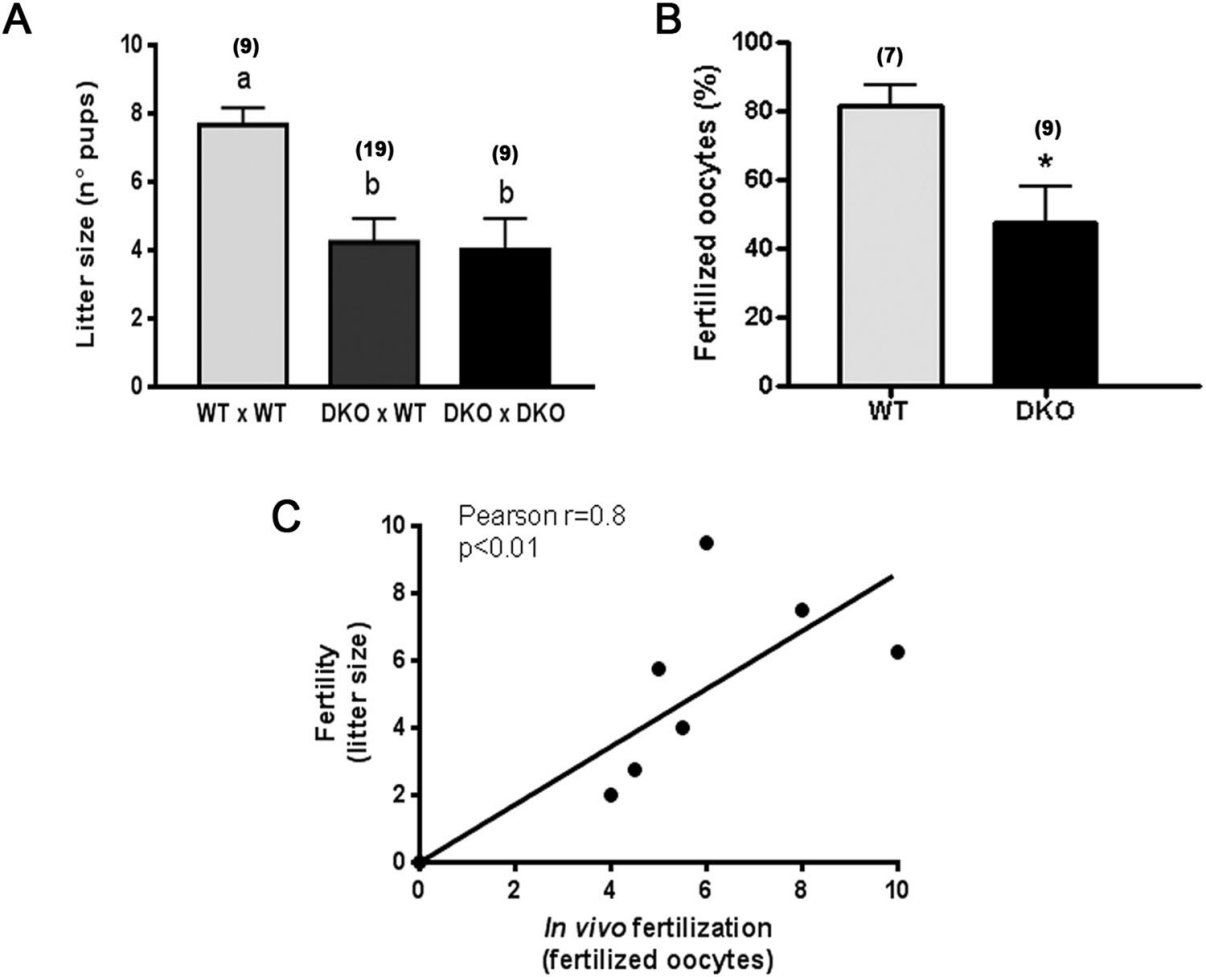
Effect of the lack of CRISP1 and CRISP4 on male fertility and *in vivo* sperm fertilizing ability: (**A**) WT or DKO adult males were bred with control females for 4 days and the number of pups was analyzed. Data are mean ± SEM; the number of males analyzed (*n*) is specified in brackets. Different letters indicate significant differences between groups (a vs b, *p* < *0.01*). (**B**) WT and DKO males were mated with natural estrous females and the percentage of fertilized eggs recovered from the ampulla was evaluated the following day. Data are mean ± SEM; the number of males analyzed (*n*) is specified in brackets. *p < *0.05*. (**C**) Correlation between the number of pups and the number of fertilized eggs recovered from the ampulla corresponding to females mated by the same males; *n* = 9, *p < *0.01*.

To investigate whether the simultaneous absence of CRISP1 and CRISP4 was capable of affecting animal fertility, wild type (WT) or DKO adult males were caged with control females for 4 days and the number of delivered pups was analyzed. Results revealed that mice lacking the two CRISP proteins simultaneously exhibited a significant decrease in their fertility in terms of average litter size (Fig. 1A). No further decrease in fertility was observed when DKO males were bred with DKO females (Fig. 1A), indicating that the absence of CRISP1 and CRISP4 has effects only in the males.

Considering the reported roles of both CRISP1 and CRISP4 in different stages of fertilization, we next investigated whether the observed fertility defects could be caused by deficiencies in the fertilization process. WT and DKO males were mated with natural estrous females, and the percentage of fertilization in the oviduct evaluated the following day. In spite of the presence of sperm in the utero of all mated females analyzed, a marked reduction in fertilization levels was observed for DKO males (Fig. 1B), suggesting a failure of ejaculated sperm to reach and/or interact with the egg. Moreover, the number of fertilized eggs recovered from estrus females mated by DKO males significantly correlated with the number of pups born from females mated by the same males (Fig. 1C) and genotyping of the pups revealed a Mendelian distribution for the genes (Fig. S3). Together, these observations suggest *in vivo* fertilization defects as responsible for the impaired fertility of DKO males.

**Figure 2:**
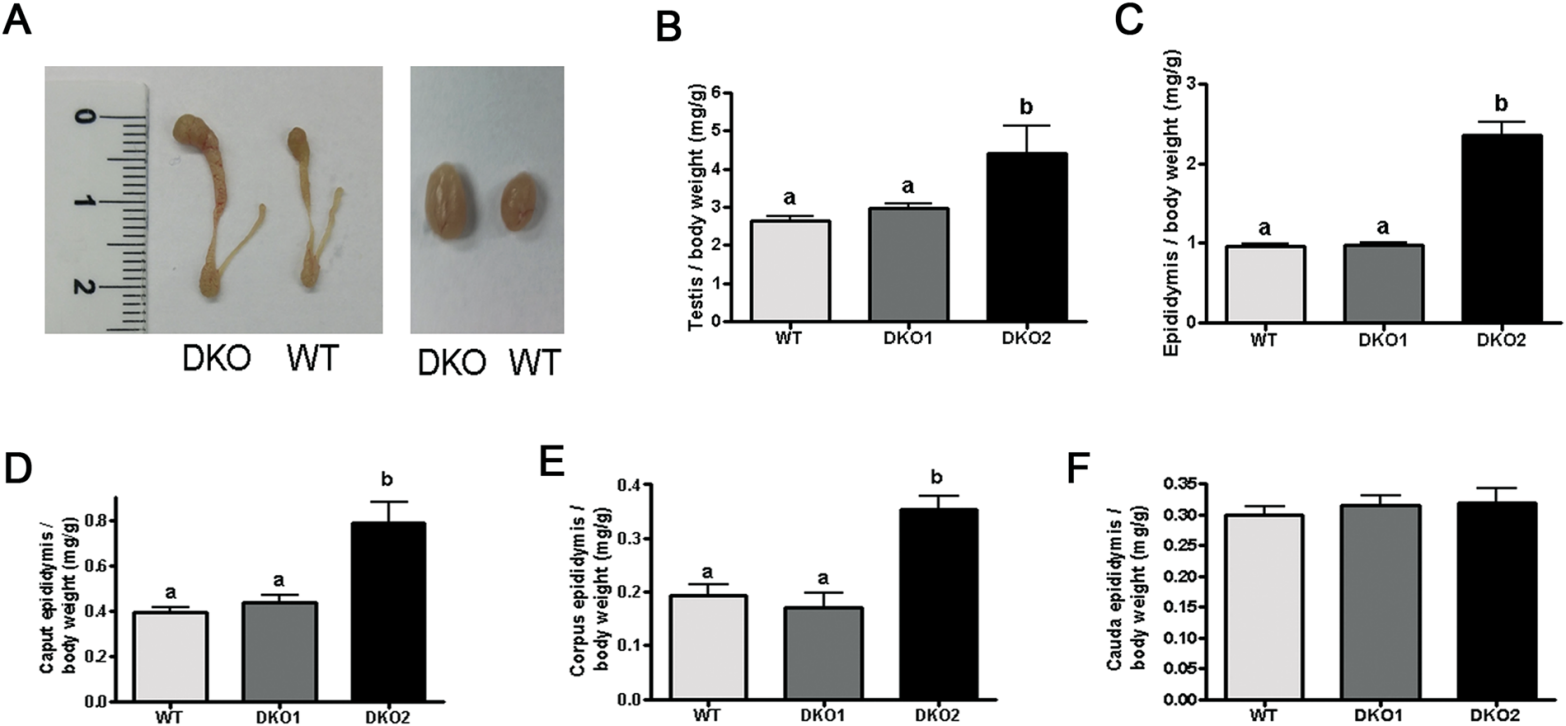
Testicular and epididymal phenotype in DKO mice: (**A**) representative pictures of DKO and WT epididymides (left) and testes (right). Testicular (**B**) and epididymal (**C**) weight relative to total body weight from WT, Group 1 (DKO1) and Group 2 (DKO2) mice. Data are mean ± SEM, *n* = 21. Caput (**D**), corpus (**E**) and cauda (**F**) weight relative to total body weight from WT, DKO1 and DKO2. Data are mean ± SEM, *n* = *9*. In all cases, different letters indicate significant between groups (a vs b, *p* < *0.01*).

To explore the mechanisms leading to the *in vivo* fertilization defects, we then decided to analyze different sperm functional parameters. Interestingly, when the testes and epididymides were removed for sperm collection, we observed that more than one third of the DKO males (30 out of the 76) had clearly bigger epididymides and testes compared to controls (Fig. 2A) either in one (20 out of 30) or both sides of the animal (10 out of 30). Based on this, DKO mice were divided into two groups: Group 1, showing epididymides and testes not different from controls, and Group 2 exhibiting bigger reproductive organs either unilateral- or bilaterally. These visible differences in the size of the organs were confirmed by the significant increase in testicular (1.7 fold) and epididymal (2.5 fold) weight of mutant mice compared to controls (Fig. 2B,C). Analysis of the different regions of the epididymis showed that whereas the weight of the caput and corpus regions were significantly higher than controls (2.01 and 1.83 fold, respectively), no differences were observed for the cauda region (Fig. 2D–F). To analyze whether this phenotype was influenced by the age of the animals, we next examined the reproductive organs from DKO males of different ages ranging from 30 days to 1 year. Results showed that whereas the phenotype was not observed in pre-pubertal mice, it was already present in 60 days-old mice at levels that did not change with the increasing age of the animals (Fig. S4).

**Figure 3:**
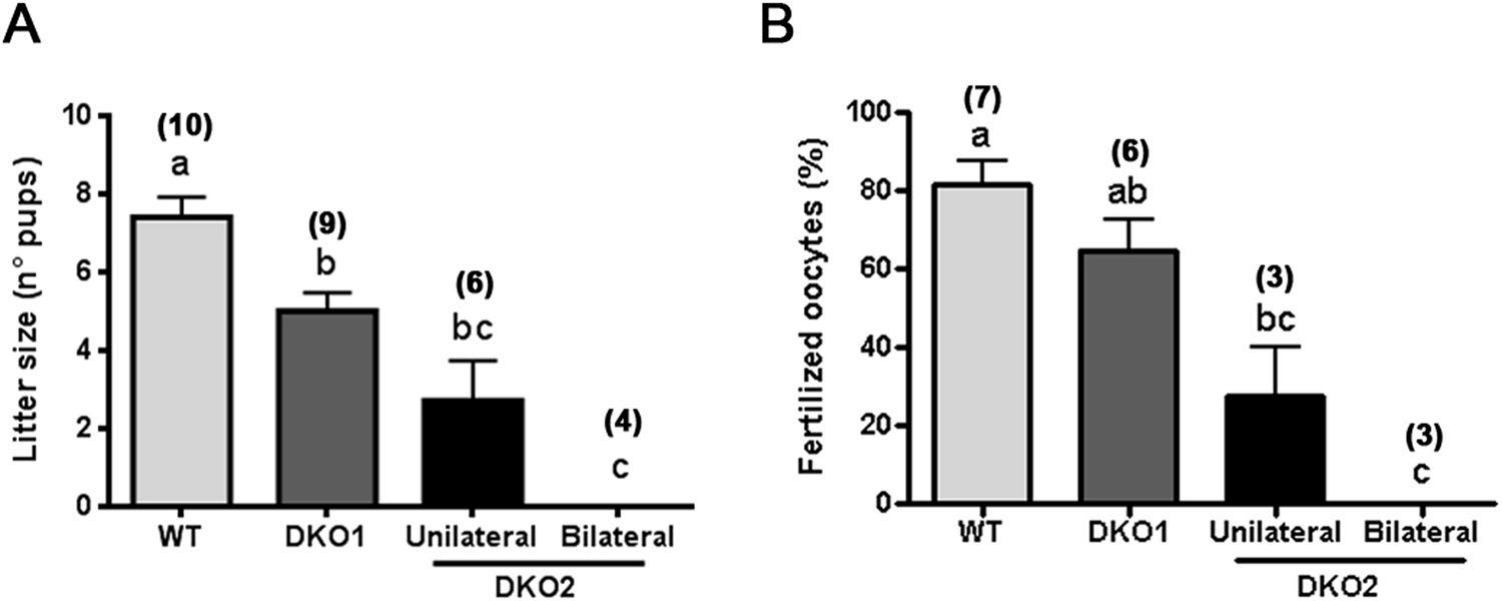
Fertility and *in vivo* sperm fertilizing ability in each DKO group: (**A**) WT males as well as DKO mice from Group 1 (DKO1) and from Group 2 (DKO2) were bred with control females for 4 days and the number of pups was analyzed. (**B**) The same males were mated with natural estrus females and the percentage of fertilized eggs recovered from the ampulla was evaluated the following day. Data are mean ± SEM; the number of males analyzed (*n*) is specified in brackets. Means not sharing a same letter are significantly different. a vs b, *p* < *0.01;* b vs c and a vs c *p* < *0.0001*.

Having established the existence of two groups of males within the DKO colony, we next re-analyzed fertility and *in vivo* fertilization outcome as a function of these groups. Our results revealed that males from both groups had a significantly reduced fertility. However, whereas mice from Group 1 exhibited a moderate subfertility, those from Group 2 showed a severe reduction in their fertility or were completely infertile when unilaterally or bilaterally affected, respectively (Fig. 3A). As previously observed, fertility levels in each group correlated with the corresponding *in vivo* fertilization rates (Fig. 3B).

### The lack of CRISP1 and CRISP4 affects the sperm fertilizing ability

**Figure 4:**
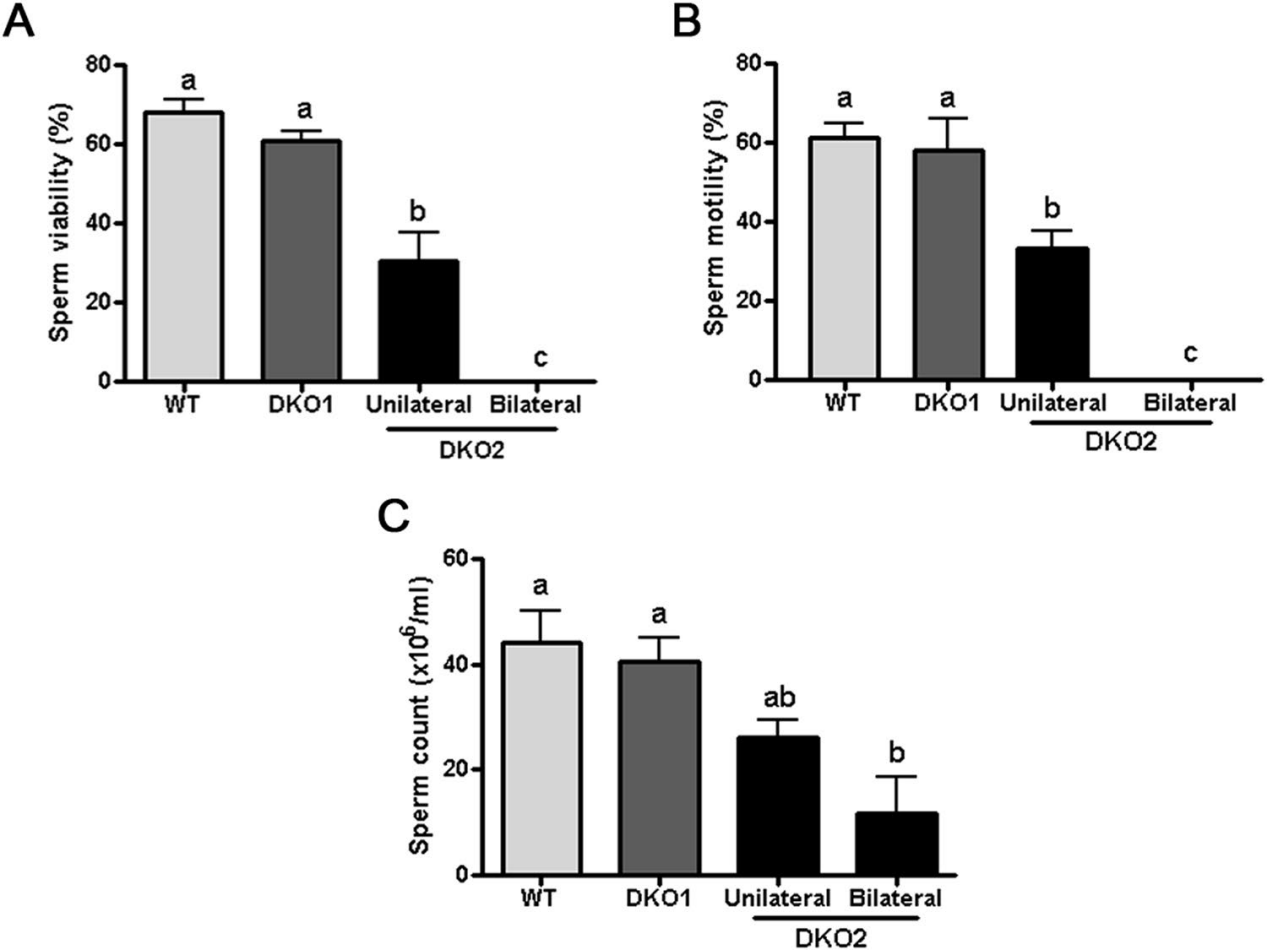
Effect of the lack of CRISP1 and CRISP4 on fresh sperm parameters. (**A**) sperm viability was determined by analyzing eosin-labelled cauda epididymal sperm under a light microscope (x400). (**B**) progressive motility was analyzed in fresh cauda epididymal sperm under light microscope (x400). (**C**) sperm count was determined using a Neubauer chamber over media containing cauda epididymal sperm obtained by swim out. Data are mean ± SEM, *n* = 10. Means not sharing a same letter are significantly different. a vs b, *p* < *0.05;* b vs c and a vs c *p* < *0.0001*).

We next analyzed several functional parameters in cauda epididymal sperm recovered from each group. Results showed that whereas sperm from Group 1 were morphologically normal and as viable as WT cells, those from Group 2 had either a significantly reduced or null viability (with detached heads and tails) when collected from males affected in one or both epididymides, respectively (Fig. 4A). Sperm motility was also significantly lower for Group 2 sperm compared to Group 1 and WT cells (Fig. 4B) but this reduction seemed to be due to viability defects. Consistent with the lower sperm motility/viability, a significantly lower number of sperm was recovered from the cauda epididymis of Group 2 males (Fig. 4C).

**Figure 5:**
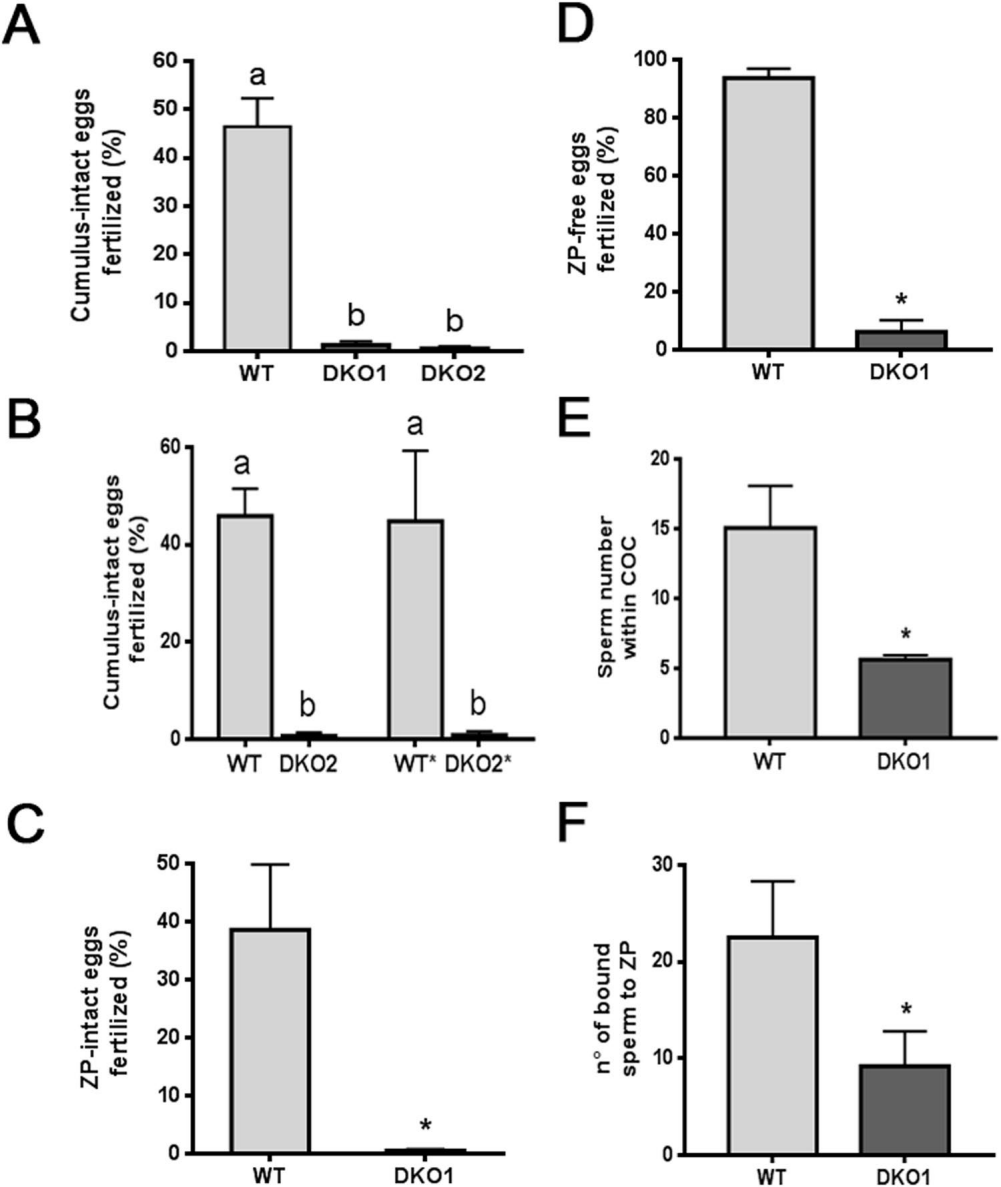
Effect of the lack of CRISP1 and CRISP4 on i*n vitro* sperm fertilizing ability: (**A–D**) Capacitated sperm from WT or DKO Group 1 (DKO1) or Group 2 (DKO2) males were co-incubated with COC for 3 h (**A**,**B**), ZP-intact eggs for 3 h (**C**) or ZP-free eggs for 1 h (**D**). At the end of all incubations, fertilization was evaluated by the presence of decondensing sperm heads within the egg cytoplasm. In (**B**) WT* corresponds to WT capacitated sperm treated to reach the low viability rates of Group 2 DKO sperm and DKO2* represents capacitated sperm from Group 2 DKO mice subjected to a swim up procedure to select viable sperm. Data are mean ± SEM, *n* = 4. Different letters indicate significant differences between groups (a vs b, *p* < *0.001*). (**E**) Hoescht-stained capacitated sperm were co-incubated with COC for 15 min and the number of sperm within the cumulus matrix was determined. Data are mean ± SEM, *n* = 4, *p < *0.05*. (**F**) Capacitated sperm were co-incubated with ZP-intact eggs for 30 min and the number of sperm bound to the ZP was evaluated. Data are mean ± SEM, *n* = 4, *p < *0.05*.

The next step was to evaluate the fertilizing ability of sperm from each group by *in vitro* fertilization using COC, ZP-intact or ZP-free oocytes to analyze different stages of gamete interaction. Because bilaterally affected DKO males had non-viable sperm, these experiments were carried out using only males from Group 1 or from unilaterally-affected Group 2. Results obtained using the same sperm concentration showed that DKO sperm from both groups were completely unable to fertilize COC (Fig. 5A), indicating that the impaired sperm fertilizing ability of mutant sperm could not just be attributed to a sperm viability defect. To further investigate this idea, WT sperm were exposed to cold media to reduce their viability to values similar to those of Group 2 (i.e. 30%) whereas sperm from Group 2 were subjected to a swim up selection procedure to reach the viability rates corresponding to control/Group1 sperm (i.e. 60%). Under these conditions, WT sperm with a low viability continued fertilizing COC normally whereas Group 2 sperm with normal viability rates remained unable to do so (Fig. 5B), indicating that the lack of fertilizing ability of Group 2 sperm was mostly due to the role of CRISP proteins in fertilization rather than to a viability defect. The following experiments aimed to analyze sperm behavior during fertilization were performed using only viable sperm from Group 1. Exposure of sperm to ZP-intact and ZP-free eggs showed significantly lower fertilization rates for mutant than for WT sperm, suggesting deficiencies at both sperm-ZP interaction and gamete fusion (Fig. 5C,D). Further analysis revealed that DKO spermatozoa showed reduced ability to penetrate the cumulus oophorus and to bind to the ZP (Fig. 5E,F), confirming that mutant sperm exhibited deficiencies at several stages of the fertilization process.

**Figure 6:**
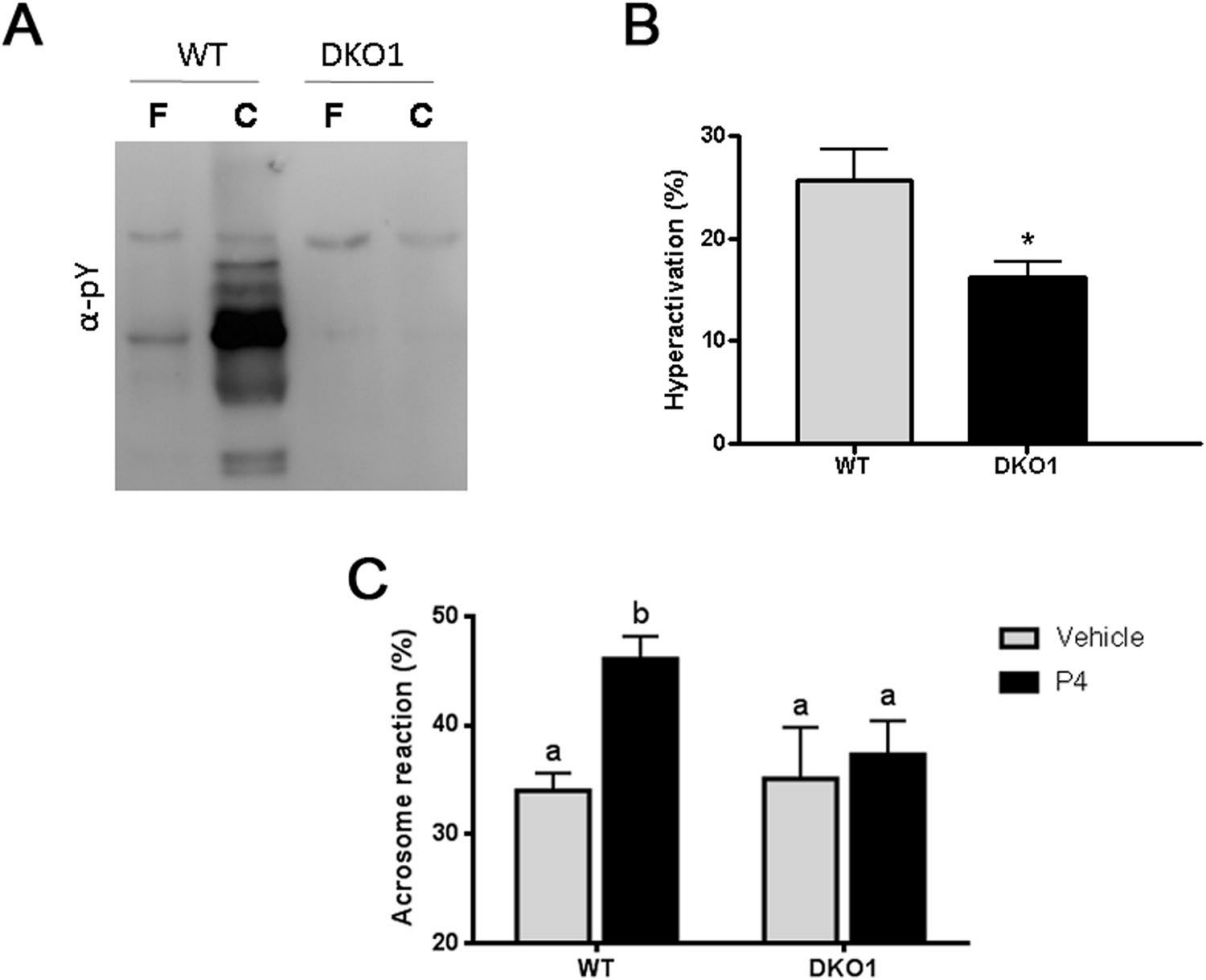
Effect of the lack of CRISP1 and CRISP4 on sperm capacitation-associated events: WT and DKO sperm from Group1 (DKO1) were incubated under capacitating conditions for 90 min and different functional parameters evaluated. (**A**) Protein tyrosine phosphorylation analyzed by western blotting using an anti-phosphotyrosine antibody (α-pY). Fresh (F) and capacitated (C) sperm were analyzed. (**A**) representative blot is shown, *n* = 4. (**B**) Percentage of hyperactivation evaluated by CASA. Data are mean ± SEM, *n* = 6, *p < *0.05*. (**C**) Percentage of acrosome reaction determined by Coomassie Brilliant Blue staining in sperm exposed to progesterone (P4) or dimethyl sulfoxide alone (vehicle) during the last 15 min of incubation. Data are mean ± SEM, *n* = *10*. Different letters indicate significant differences between groups (a vs b, *p* < *0.01*).

To better understand the mechanisms leading to these *in vitro* fertilization defects, several capacitation-associated sperm parameters were evaluated in Group 1 sperm. Western blot analysis revealed a failure of mutant sperm to undergo the characteristic increase in protein tyrosine phosphorylation levels that occurs during capacitation (Fig. 6A) whereas Computer-assisted sperm analysis (CASA) showed a significant reduction in the total percentage of hyperactivation of mutant sperm compared to controls (Fig. 6B). Finally, analysis of the percentage of both spontaneous and progesterone-induced acrosome reaction at the end of the incubation showed a significant reduction in the ability of DKO sperm to respond to the hormone compared to controls (Fig. 6C).

### The lack of CRISP1 and CRISP4 leads to immunological deregulation within the reproductive tract

**Figure 7:**
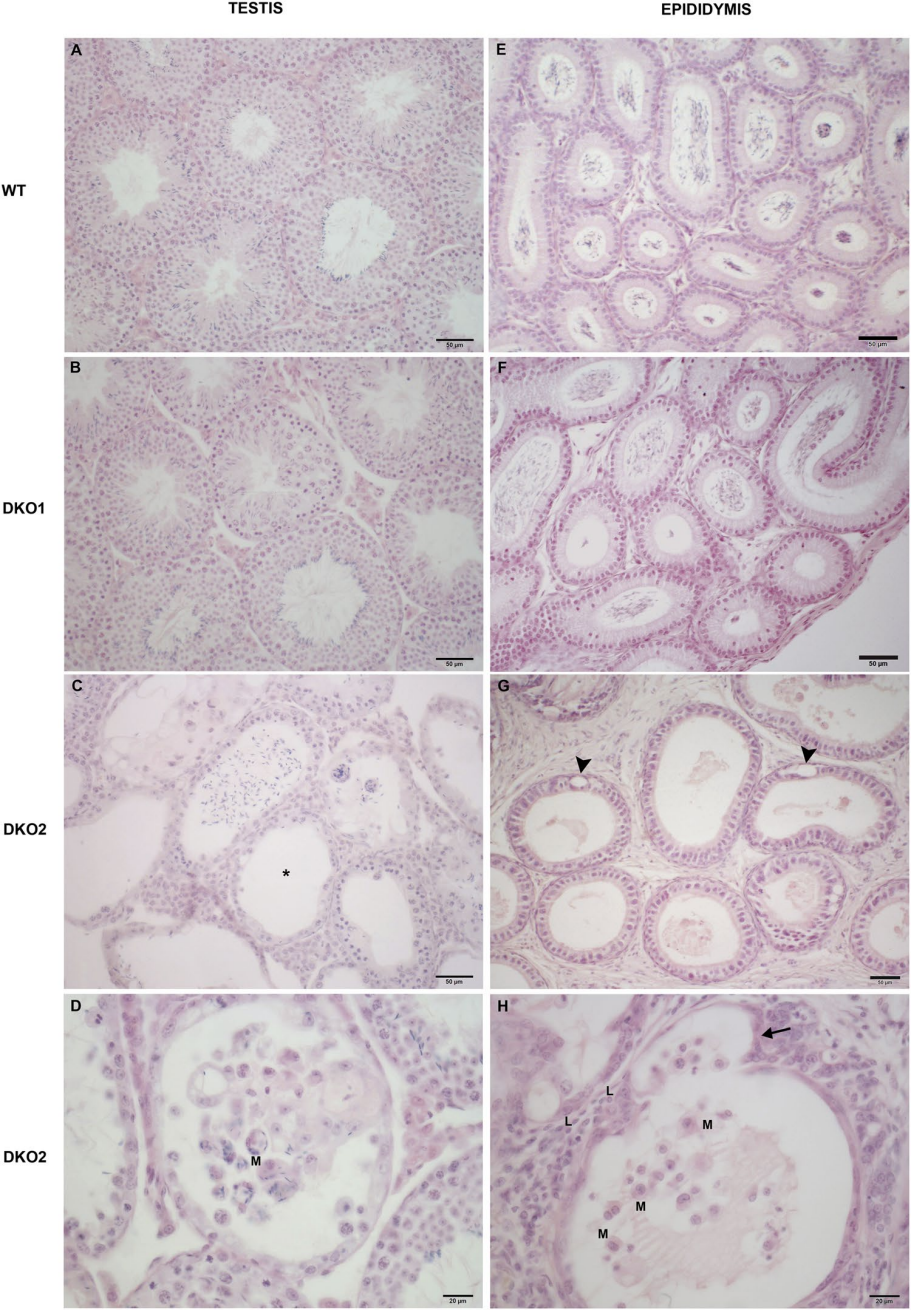
Histopathology of DKO testes and epididymides: Representative microphotographs of paraffin testis (**A–D**) and distal caput epididymis (**E–H**) sections from WT, Group 1 (DKO1) and Group 2 (DKO2) DKO mice stained with hematoxylin-eosin. Normal histology is seen in WT mice (**A**,**E**) and DKO1 mice (**B**,**F**). In contrast, testis and epididymis sections from DKO2 mice exhibited lymphomononuclear cell infiltrate in lumen and insterstitium (**C**,**D**,**H**) (L: lymphocytes and M: macrophages). Foci of seminiferous tubules severely damaged showing sloughing of germ cells, aspermatogenesis (asterisk) and atrophy (**C**). In epididymis of DKO2 mice, damage is represented by epithelial cytoplasmic vacuolization (**G**, arrowheads) and thinning of the epididymal epithelium in a tubule also showing evaginations (**H**, arrow) In all cases, DKO2 correspond to the enlarged organs from either unilateral- or bilaterally affected mice.

**Figure 8:**
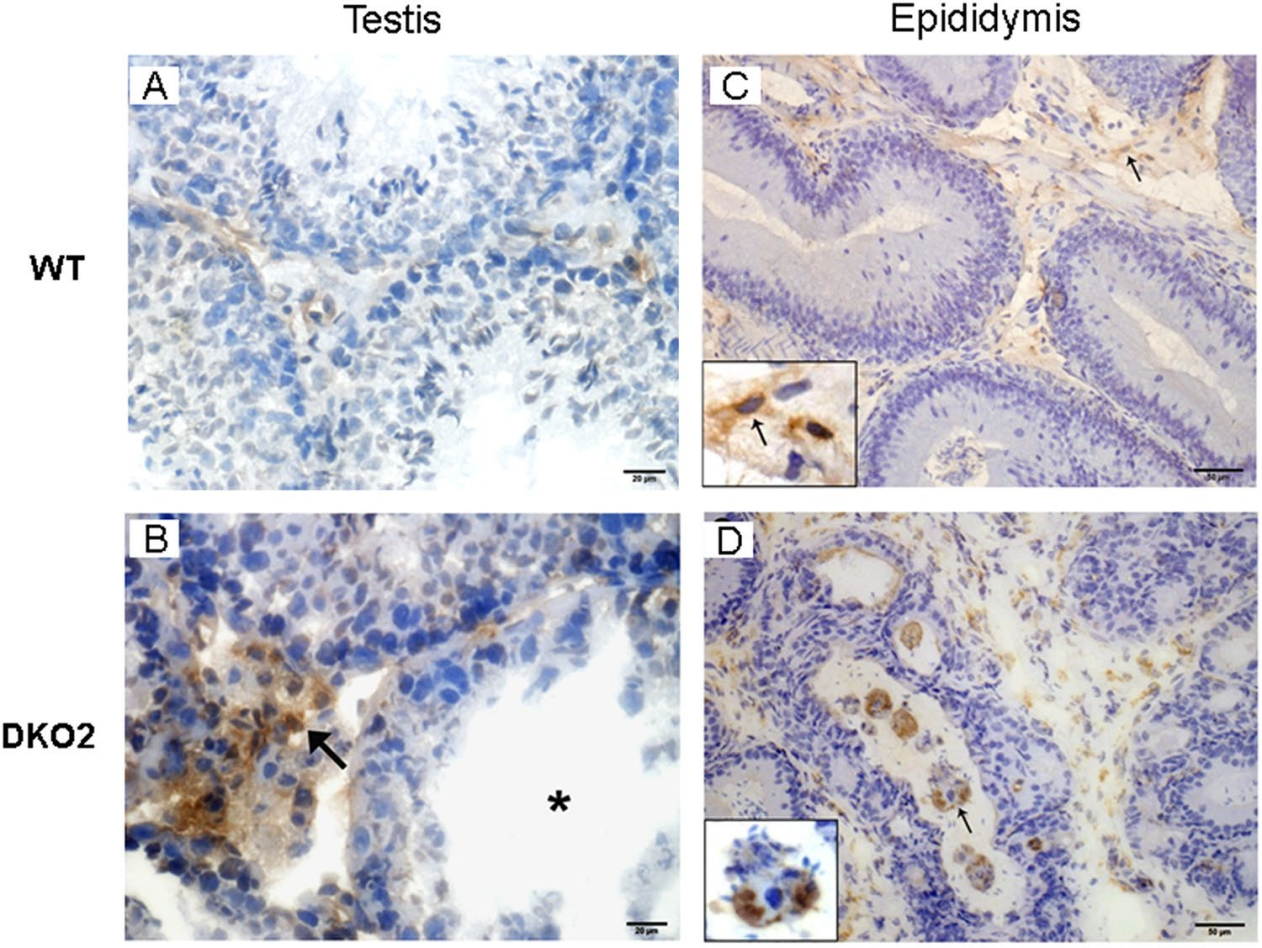
Analysis of macrophages in testis and epididymis: Immunoperoxidase technique using F4/80 antibody to detect macrophages was applied to cryostat testis (**A**,**B**) and epididymis (**C**,**D**) sections from WT mice, or DKO mice from Group 1 (DKO1) or Group 2 (DKO2). Similar results were observed between WT and DKO1 mice. Representative images of WT mice (**A**,**C**) show scarce number of F4/80+ cells scattered in the interstitium. In contrast, DKO2 mice (**B**,**D**) show numerous F4/80+ cells in the testis interstitium (**B**, arrow) close to a damaged seminiferous tubule (**B**, asterisk) and in the lumen of epididymal tubules with spermatozoa heads within the macrophage cytoplasm (**D**, arrow). DKO2 correspond to the enlarged organs from either unilateral- or bilaterally affected mice.

As an approach to better understand the phenotypes observed for both Group 1 and Group 2 DKO mice, the testes and epididymides were subjected to histopathological evaluation. Results revealed that both tissues from Group 1 were not different from WT (Fig. 7A,B,E,F) but those from Group 2 presented pathological features. Whereas the testes exhibited foci of aspermatogenic and atrophic seminiferous tubules (Fig. 7C,D), all epididymal regions revealed impairment of the epithelium represented by cell cytoplasm vacuolization and evaginations that were more severe in caput (Fig. 7G,H). In addition to this, the testes and epididymides from Group 2 showed lymphomononuclear cells within the interstitium and tubular lumen (Fig. 7C,D,G,H). Further examination by immunohistochemistry confirmed that both organs of Group 2 mice exhibited an increased presence of macrophages in the interstitium and in the tubules (Fig. 8B,D), some of them phagocyting sperm heads within the epididymal lumen (Fig. 8D). Together, these observations indicate that males from Group 2 exhibited tissue inflammation and damage of seminiferous and epididymal epithelia corresponding to an epididymo-orchitis.

**Figure 9:**
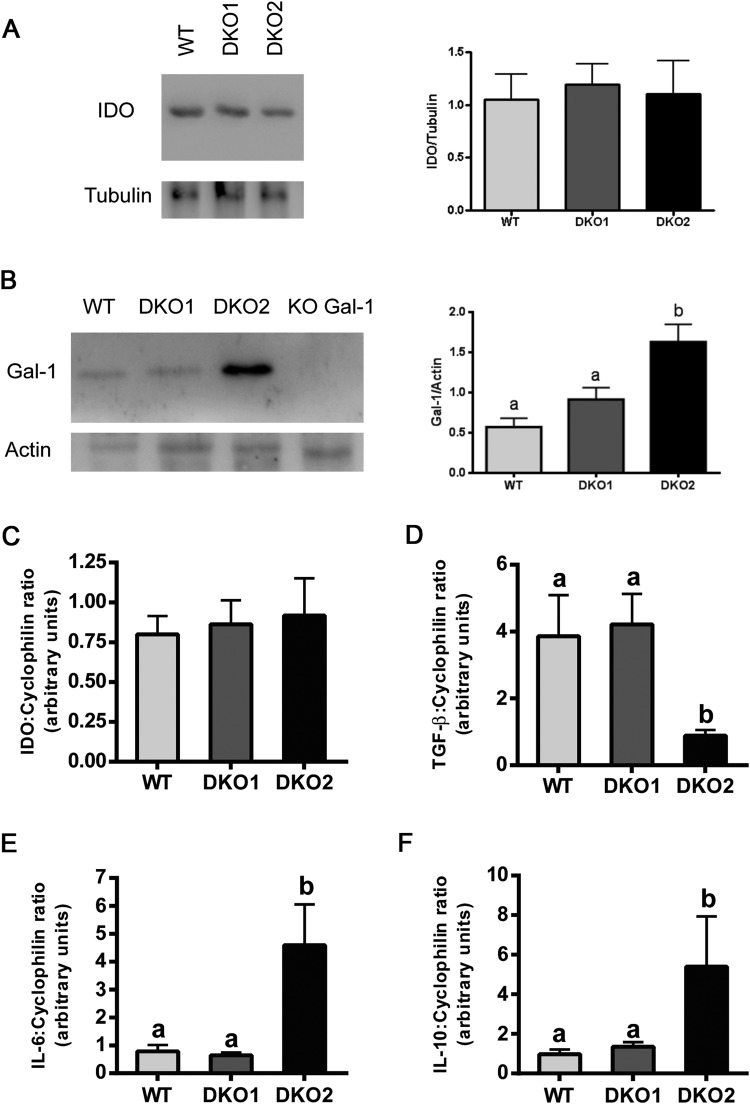
Analysis of molecules associated with immune-inflammatory response in DKO epididymides: Expression of immunomodulator proteins IDO (**A**) and Gal-1 (**B**) were analyzed by western blotting in epididymides from WT, Group 1 (DKO1) and Group 2 (DKO2) males and relativized to housekeeping proteins. Expression of genes corresponding to different pro- and anti-inflammatory molecules IDO (**C**), TGF-β (**D**), IL-6 (**E**) and IL-10 (**F**) were evaluated by RT-PCR in epididymides from WT, Group 1 (DKO1) and Group 2 (DKO2) males and relativized to cyclophilin expression. Data are mean ± SEM, *n* = 6. Different letters indicate significant differences between groups (a vs b, *p* < *0.05*). In all cases, DKO2 values correspond to the inflamed organs from unilateral- or bilaterally affected mice. Full-length blots/gels are presented in Supplementary File.

Considering the epididymal origin of the two lacking proteins, we further investigated the immunological state within the epididymis of both Group 1 and Group 2 DKO mice by analyzing the expression level of several molecules associated with inflammatory responses. RT-PCR and western blot results showed that the levels of IDO, an anti-inflammatory and immunosuppressive enzyme abundantly expressed in the epididymis, were not significantly different between groups (Fig. 9A,C). In addition to this, we also analyzed the levels of Gal-1, a pleiotropic lectin involved in the modulation of immune response and recently described to be expressed by the epididymis in mice^35^. In this case, we observed significantly higher levels of Gal-1 by Western blot in Group 2 compared to Group 1 and WT (Fig. 9B). Analysis of the expression of cytokines within the epididymis by RT-PCR showed that whereas TGF-β levels were significantly lower in Group 2 than in WT mice (Fig. 9D), expression of IL-6 and IL-10 was upregulated in Group 2 compared to controls (Fig. 9E,F). No differences were found for any of these cytokines between Group 1 and WT, suggesting the existence of a molecular disbalance of the epididymal immunotolerant state only in Group 2 mice.

### The lack of CRISP1 and CRISP4 affects epididymal epithelium differentiation

**Figure 10:**
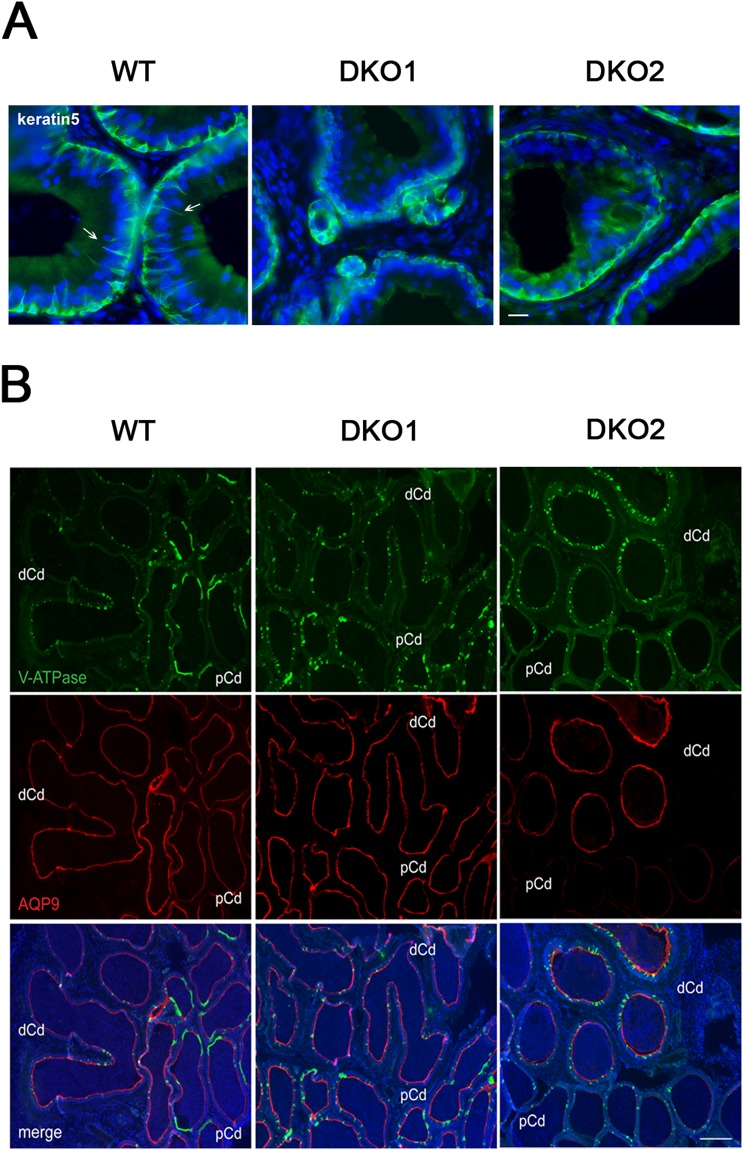
Effect of the lack of CRISP1 and CRISP4 on epididymal epithelium cells: (**A**) Immunolabeling of keratin 5 (green), a marker of basal cells, in the initial segment of WT, and DKO from Group 1 (DKO1) and Group 2 (DKO2) epididymides. Basal cells in WT tissues present luminal reaching axiopodia (arrows) not observed in DKO groups. Nuclei are labeled with DAPI (blue). Scales correspond to 10 µm. (**B**) Double immunolabeling of V-ATPase B1 subunit, a marker of clear cells (green) and AQP9, a marker of principal cells (red) in the cauda epididymis of WT and DKO males. AQP9 staining was restricted to the apical membrane of principal cells (merge image). Clear adjacent cells showed positive staining to V-ATPase in the apical membrane only in WT (merge image). Nuclei are labeled with DAPI (blue). pCd: proximal cauda; dCd: distal cauda. DKO2 correspond to the enlarged organs from unilateral- or bilaterally affected mice. Scales correspond to 100 µm. Representative images are shown.

**Figure 11:**
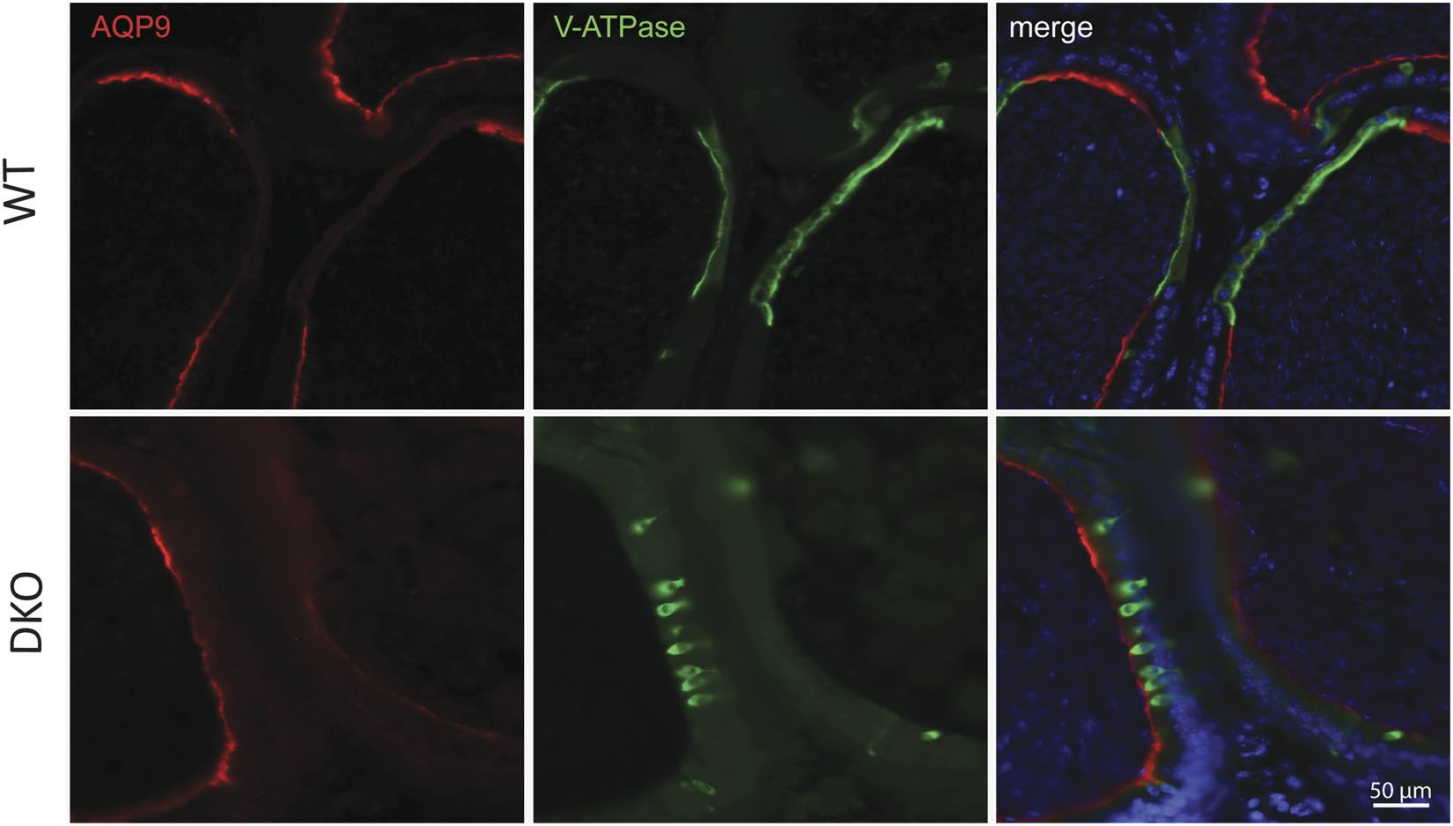
Effect of the lack of CRISP1 and CRISP4 on cauda epididymal epithelium. Double immunolabeling of AQP9, marker of principal cells (red) and V-ATPase B1 subunit, marker of clear cells (green) in the proximal cauda epididymis of WT and DKO males. Note the more packed together and immature narrow phenotype of clear cells in DKO (similar to pre-pubertal mice) and the absence of the characteristic rows of clear cells observed in WT. Nuclei are labeled with DAPI (blue). Representative images are shown. DKO2 correspond to the enlarged organs from either unilateral- or bilaterally affected mice.

Based on the involvement of the epididymal epithelium in the regulation of the immune environment, the different cell types that comprise this pseudostratified epithelium (i.e. basal, clear, narrow, principal cells) were analyzed in mice from both groups by immunofluorescence. Studies using an antibody against keratin5, a marker of basal cells, revealed that whereas the initial segment (IS) of WT mice exhibited the characteristic basal cell projections towards the lumen known as axiopodia^36^, very few or no projections were detected in the IS of Group 1 or Group 2 males (Fig. 10A). Whereas in the caput and corpus regions there was a reduction in AQ9 staining (principal cells) in both groups as well as in V-ATPase staining (clear cells) in Group 2 (Fig. S5), in the cauda region of both groups, clear cells were more packed together and exhibited an immature narrow phenotype compared to WT (Figs 10B and 11), similarly to what is observed in a pre-pubertal animal. In addition, the proximal cauda epididymal epithelium of both groups did not exhibit the characteristic rows of clear cells observed in WT mice (Figs 10B and 11). While similar levels of AQP9, a marker of principal cells, were observed in the distal cauda of all groups, no AQP9 expression was detected in the proximal cauda in Group 2 mice (Fig. 10B). Together, these observations confirmed the presence of epididymal epithelium defects in mice from both groups with a stronger phenotype in males from Group 2.

**Figure 12:**
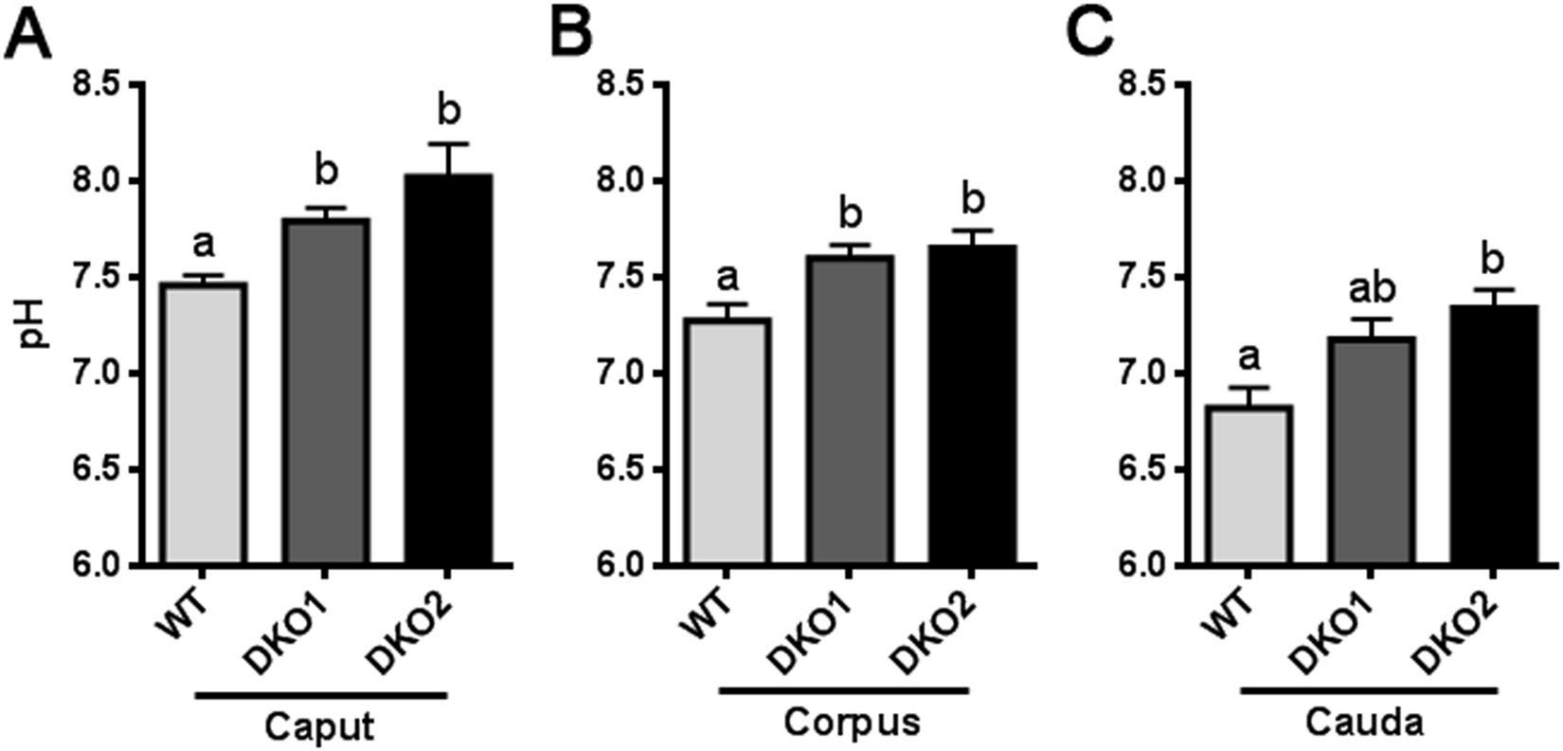
Effect of the lack of CRISP1 and CRISP4 on epididymal luminal pH: Epididymal pH was measured in the luminal content of caput (**A**), corpus (**B**) and cauda (**C**) epididymal regions of WT mice and DKO males from Group 1 (DKO1) or from Group 2 (DKO2). DKO2 values correspond to the inflamed organs from unilateral- or bilaterally affected mice. Data are mean ± SEM, *n* = *6*. Means not sharing a same letter are significantly different. a vs b, *p* < *0.01;* b vs c and a vs c *p* < *0.0001*.

Considering the role of the epididymal epithelium in maintaining the proper luminal pH for sperm maturation, we next measured intraluminal pH in different regions of the epididymis. As shown in Fig. 12A–C, there was a significant increase in luminal pH in both Group 1 and Group 2 epididymides compared to controls, supporting the idea that the lack of CRISP1 and CRISP4 was affecting the epididymal epithelium and, thus, its involvement in the luminal pH acidification required for proper sperm maturation and storage.

## Discussion

CRISP1 and CRISP4 are expressed in high concentrations in the epididymis where they associate with sperm plasma membrane. Although sperm lacking either CRISP1 or CRISP4 have strong deficiencies in fertilizing eggs *in vitro*^29–32^, knockout mice for each of these proteins are fertile, suggesting the existence of compensatory mechanisms among CRISP family members. Our present observations showing that males lacking both CRISP1 and CRISP4 exhibit severe fertility defects confirm this hypothesis and reveal the relevance of CRISP proteins for animal fertility. Our results are consistent with several reports showing that knockout mice for individual proteins are fertile but exhibit fertility defects when other proteins are absent as well. Male mice lacking the genes *Tnp2*, *Acr*, *H1.1*, *H1t* or *Smcp* individually are fertile whereas several triple knockout mice for these genes exhibit drastic reductions in male fertility^37^. As sperm are transcriptionally inactive cells, numerous proteins associate with them during their transit through the male and female reproductive tracts to regulate their fertilizing ability leading to cooperative and compensatory mechanisms to guarantee sperm functionality.

The significant correlation observed between the number of born pups and fertilized oviductal eggs in females mated by the same males indicates that fertility defects in DKO males were due to deficiencies in the *in vivo* fertilization process. This conclusion was further supported by the finding that fertility also correlated with the *in vivo* fertilization results when analyzed as a function of the different DKO subgroups i.e. a major group with normal testes and epididymides (Group 1) and a minor group showing clear signs of inflammation (i.e. epididymo-orchitis) in the two organs (Group 2) either in one or both sides. As CRISP1 and CRISP4 are expressed in the epididymis but not in the testes, testicular defects might have appeared as a consequence of the alterations in epididymal physiology as observed in men where epididymitis also affects the testes to elicit a combined epididymo-orchitis^38^. Our studies using animals of different ages show that the inflammatory phenotype appears after puberty and remained at similar levels thereafter, coincident with the appearance of CRISP proteins^10,39^ and consistent with the concept that development of the epididymo-orchitis phenotype occurs with the appearance of sperm antigens within the reproductive tract^40,41^. In this regard, the observation that fertility and fertilization rates in males with inflamed organs correlated with the percentage of viable sperm in these mice supports sperm viability defects as main responsible for their fertility impairment. Interestingly, fertility was significantly reduced also in those males with normal reproductive organs and normal sperm viability supporting the roles of CRISP proteins in fertilization^19,20,30,33,34^ as relevant factors for male fertility.

*In vitro* fertilization assays to evaluate changes in the sperm fertilizing ability that could explain the reduced fertilization/ fertility rates in DKO males showed that both Group 1 and Group 2 sperm were unable to fertilize COC, confirming the existence of sperm fertilizing deficiencies that did not rely on the viability of the cells. This was further supported by the finding that those viable sperm selected from Group 2 remained infertile when exposed to COC. Additional *in vitro* fertilization experiments exposing viable sperm from Group 1 to eggs surrounded or not by their coats confirmed that these mutant sperm exhibited lower ability to penetrate the cumulus, to bind to the ZP and to fuse with the egg, indicating the existence of sperm defects at different stages of the fertilization process. The impaired sperm-ZP binding and gamete fusion might result from the lack of sperm CRISP proteins known to be involved in these two stages of fertilization through their interaction with egg complementary sites^19,20,33,34^. Nevertheless, the finding that none of COC and ZP-intact eggs showed accumulation of sperm in the perivitelline space supports severe deficiencies at steps previous to gamete fusion. Further studies revealed that these deficiencies might be due to a failure of mutant sperm to undergo a normal capacitation process as judged by the significant reduction in several capacitation-associated sperm parameters such as protein tyrosine phosphorylation, progesterone-induced acrosome reaction and hyperactivation. Whereas defects in hyperactivation might be responsible for the lower levels of cumulus- and ZP-penetration known to require this vigorous sperm motility^24,42^, the lower levels of induced-acrosome reaction might affect the sperm ability to penetrate the ZP which absolutely depends on the occurrence of this exocytotic event. Moreover, considering recent findings supporting that most sperm undergo the acrosome reaction in the isthmus^43,44^, we cannot exclude the possibility that the lower acrosome reaction rates also contribute to the reduced levels of cumulus penetration.

The final phenotypes observed for each functional parameter in DKO mice seem to be a combination of the phenotypes corresponding to the single KO for each CRISP protein (Table 1). Thus, whereas in some cases only one of the proteins contributed to the DKO phenotype (i.e. CRISP4 for protein tyrosine phosphorylation and CRISP1 for hyperactivation and for ZP binding) in others, both proteins seem to do so (i.e. progesterone induced-acrosome reaction, gamete fusion) or to have a synergistic effect (i.e. cumulus penetration). This accumulation of functional defects may explain why DKO sperm exhibit *in vivo* fertilization defects not observed for the single mutant sperm, supporting the idea that the beneficial effects of the female reproductive tract may mask sperm functional defects that are detected under more demanding conditions as previously reported^45^.

**Table 1:** Comparison between the reproductive phenotypes of individual (CRISP1 or CRISP4) KO and DKO mice.

	*Crisp1* ^−/−^	*Crisp4* ^−/−^	DKO
Tyrosine phosphorylation	=	Affected	Affected
P4-induced acrosome reaction	Affected	Affected	Affected
Hyperactivity	Affected	=	Affected
Cumulus penetration	=	=	Affected
ZP binding	Affected	=	Affected
Fusion ability	Affected	Affected	Affected
*In vitro* fertilization	Affected	Affected	Affected
*In vivo* fertilization	=	=	Affected
Fertility	=	=	Affected

= and “affected” refer to no significant or significant differences from the corresponding WT controls, respectively.

Histopathological and immunohistochemical studies of the testes and epididymides aimed to investigate the mechanisms underlying DKO sperm functional defects revealed that Group 2 mice exhibited tissue damage and a clear immune response. These phenotypes were neither observed in Group 1 nor in the single KOs for each protein, supporting the relevance of both CRISP1 and CRISP4 for normal epididymal function and the existence of compensatory mechanisms between the two molecules. The finding that Group 2 epididymal tissues exhibited altered levels of the immunomodulator Gal-1 as well as of several cytokines such as Il-6, Il-10 and TGF-β supports a disruption of the characteristic immunological tolerance of the epididymis associated with epididymitis^46^. Although these molecular alterations were not observed in Group 1, we cannot exclude possible changes in other non-analyzed mediators of inflammation. Also, given that CRISP are present in organs with immunological roles (i.e. thymus, spleen)^47^ and have high homology with proteins known to be involved in host defense^12^, it cannot be ruled out that CRISP1 and CRISP4 play immunoregulatory roles that contribute to the establishment of the particular tolerant environment within the epididymis. Consistent with this, cytokine changes similar to those observed in Group 2 mice have also been observed in the epididymis from mice lacking IDO, an immunosuppressive molecule abundantly expressed in the epididymis^48^. The potential roles of CRISP as immunoregulators are currently under investigation.

To gain insights into the mechanisms that trigger the immunological response in DKO mice, we next analyzed the state of the epididymal epithelium known to participate in the immune control of the organ^41^. Interestingly, immunofluorescence studies revealed defects in all epithelial cell types in DKO mice with a more severe phenotype in Group 2. The observation that basal cells in the IS lack their characteristic axiopodia^36,49^, that principal and clear cells from all regions express less AQP9^50^ and V-ATPase, respectively, and that clear cells of the proximal cauda exhibit an immature “narrow” and “packed” phenotype^51^ indicates the relevance of CRISP1 and CRISP4 for a proper epididymal epithelium differentiation. In agreement with our observations, mice lacking RNaseIII enzyme Dicer1 in the epididymis and expressing lower levels of both CRISP1 and the androgen receptor, also possess an undifferentiated state in their epididymal epithelium resembling that of pre-pubertal mice^52^. As the regulatory mechanisms underlying epididymal epithelial cell differentiation during puberty are still unclear, these results will certainly contribute to a better understanding of this critical process.

Defects in epididymal epithelium differentiation could well explain the increase in luminal pH observed in DKO from both groups. In this regard, DKO mice exhibit defects in clear cells which have an active role in luminal acidification as well as in principal cells involved in bicarbonate reabsorption and proton secretion^53^. This, together with the lack of basal cell projections capable of sensing the luminal content and regulating clear cell activity may affect the complex intercellular network that controls luminal acidification. Moreover, considering that luminal acidification involves an increase in cAMP concentration in both clear and principal cells^54^, luminal pH defects in DKO mice might be associated with the recently reported ability of CRISP1 to regulate the intracellular cAMP signaling cascade^31^. A luminal pH increase was also detected in the epididymis of infertile *C-ros*^*−/−*^ mice as consequence of a failure in pubertal differentiation of the IS^55^ and in epididymides lacking Foxi1, an important regulator of gene expression, including the proton pumping V-ATPase, in narrow and clear cells^56^. The observed increase in luminal pH might be responsible for the impaired sperm fertilizing ability observed in Group 1 as it is known that luminal pH contributes to create the proper environment that maintains sperm quiescent during sperm maturation and storage in the epididymis^51,54^. In addition, given the reported ability of CRISP1 and CRISP4 to inhibit CatSper and TRPM8 activity, respectively^21,29^, the lack of these proteins might also directly affect sperm intracellular calcium levels leading to the different functional defects observed in DKO spermatozoa.

The finding that both epithelium and luminal acidification defects are present in mice that do not exhibit inflammatory phenotype indicates that these defects do not occur as a consequence of the immune reaction. In Group 2, however, an additional effect of the immune response on the epithelium integrity cannot be excluded. As epithelial cells are involved in maintaining the blood epididymal barrier (BEB), an anatomical, physiological and immunological barrier that prevents sperm antigens from escaping the duct^57–59^, it is possible that the more severe epididymal epithelium defects observed in Group 2 compromise the structure and/or function of the BEB allowing exposure of sperm to the immune system and eliciting an immunological response. Supporting this possibility, males lacking SED-1, a sperm protein secreted by the epididymis and involved in sperm-egg interaction^60^, also exhibit defects in fertilization, fertility and epithelium integrity accompanied by the presence of spermatic granulomas^61^ characterized by breakdown of the epithelium and the BEB^62^.

How the loss of CRISP1 and CRISP4 leads to the epithelium differentiation and luminal acidification defects observed in DKO mice might be linked to the known ability of these proteins to regulate ion channels^21,29^. As both CRISP1 and CRISP4 are expressed in epithelial cells^10,11,63^, the lack of these two calcium channel regulators might affect their functionality, cross-talk and control of luminal environment. Moreover, it is possible that CRISP1 and CRISP4 regulate TRPV6, a pH dependent-Ca^2+^ channel present in epididymal principal cells and reported to have an essential role in Ca^2+^ transport across the epithelia and in Ca^2+^ homeostasis in the epididymis as well as in male fertility^64–66^. This is further supported by the reported roles of both CRISP1 and CRISP4 in TRP channel regulation^21,29^.

Although the molecular mechanisms underlying the different phenotypes observed in DKO mice remain to be elucidated, it is known that phenotypes are rarely an intrinsic property of target alleles but instead result from integration of their activities with environmental context and genetic background^67–69^. In this regard, a recent study by our group reports the influence of the genetic background on the reproductive phenotype of mice lacking CRISP1^31^ while the present study reveals differences in several functional parameters between our CRISP4 KO colony and those generated in other genetic backgrounds^29,32^. Thus, individual variabilities in the genetic background might be responsible for the different phenotypes observed in Group 1 and Group 2 DKO mice. Differences in the genetic context and environmental conditions might also explain recent observations published during the preparation of this manuscript showing the absence of fertility and *in vitro* fertilization defects in DKO mice lacking CRISP1 and CRISP4 and exhibiting sperm functional defects (i.e. motility, AR, pTyr) and immune infiltrates in the epididymal stroma of old males^70^.

Interestingly, Group 2 animals developed either unilateral or bilateral epididymo-orchitis and most of the unilateral inflammations (75%) were observed in the left side. A left bias spermatogenic failure has been also observed in Dnd1Ter/+ mice^71^ as well as in the incidence of testicular tumor^72,73^, cryptorchidism and varicocele in humans^74,75^, arising probably from physiological differences that stem from left/right asymmetry in vascular architecture.

In humans, inflammation of male genital tract is thought to be a primary etiological factor in male infertility and the incidence of epididymitis is largely more frequent than orchitis,^41,76^. Based on this, and considering that human epididymal CRISP1 has been proposed to represent the equivalent of both mouse CRISP1 and CRISP4^34^, DKO mice might become an excellent model for studying the mechanisms underlying epididymal physiology and the reported non-infectious epididymitis in men^77,78^.

Together, our studies reveal that disruption of both epididymal *Crisp1* and *Crisp4* genes affects epididymal epithelium differentiation and luminal environment leading to male fertility defects. Whereas in most of the cases, the epithelium and luminal defects specifically affected the sperm fertilizing ability, in some animals there were clear signs of an immunological reaction and severe sperm viability defects. The fact that neither the epithelium alterations nor the immunological response were observed in the single KO for each protein supports the existence of a functional cooperation and compensation between individual CRISP proteins to guarantee the success of reproduction. We believe these results will contribute to a better understanding of the fine-tuning mechanisms underlying sperm maturation and immunotolerance within the epididymis with clear clinical implications for male infertility and fertility regulation. Moreover, considering the efficient functional cooperation between epithelial and immune cells that operates in the epididymis to regulate the luminal environment, the DKO model could bring insights beyond the reproductive field that contribute to explain the functionality of other epithelia with similar cellular cross-talk and/or the extremely low incidence of cancer that exists in the epididymis.

## Materials and Methods

### Animals and Ethical Approval

Adult (2–12 months-old) males and females as well as pre-pubertal males (30 days-old) were used. For comparison among groups, animals of matching ages were used in each experiment. Animals were maintained with food and water ad libitum in a temperature controlled room with a 12:12 hour light:dark cycle. Approval for the study protocol was obtained from the Animal Care and Use Committee of the Research Institute for Microbial Diseases (Osaka University, Osaka, Japan) and the Bioethics Committee of the Institute of Biology and Experimental Medicine (CONICET, Buenos Aires, Argentina). Experiments were conducted in accordance with the Guide for Care and Use of Laboratory Animals published by the National Institutes of Health (NIH).

### Generation of CRISP4 deficient mice

The targeting vector for *Crisp4* (PRPGS00028_A_A10) (Fig. S1) was obtained from the International Knockout Mouse Consortium (https://www.mousephenotype. org/imits/targ_rep/targeted_alleles/10655) and electroporated into C57BL/6N EGR-G101 embryonic stem cells^79^ after linearization with AsiSI. Clones were then selected using G418. The correctly targeted embryonic stem cells were injected to ICR 8-cell embryos to obtain chimeric mice. These chimeric males were mated with C57BL/6*DBAF1 females and germ-line transmission was confirmed by PCR analysis. To remove the floxed-exon, F1 mice were mated with B6D2 CAG-Cre Transgenic animals that ubiquitously express Cre^80^ (Fig. S1). The obtained *Crisp4*^+/−^ animals were intercrossed to obtain *Crisp4*^*−/−*^ homozygotes. To evaluate mRNA expression, PCR was performed using mouse cDNA as the template. The following primers were used: 5′-GGAAATTGTCAATACCCATAAC-3′ and reverse 5′-TGTCACAGTACCTCGCCAAG-3′ for *Crisp4*. The expected size of the PCR product is ~100 bp for *Crisp1* and ~125 bp for *Crisp4*.

### Generation of CRISP1 and CRISP4 deficient mice

Double CRISP1/CRISP4 knockout (DKO) mice were generated by mating animals from C57BL/6 *Crisp1*^*−/−*^^31^ and C57BL/6*DBA *Crisp4*^*−/−*^ single KO. Genotyping was carried out by PCR using mouse genomic DNA as the template and the following primers: 5′-AGACAAAGAGACCACCAACAGATT-3′ and reverse 5′-AGTACAGCAGCCAAGAAGAACAG-3′, and 5′-CTACCCGCTTCCATTGCTC-3′ for *Crisp1* and 5′-ACCCTCACCTATCCTTGCTGGCAG-3′ and reverse 5′-CTTTAGAATACCATGATACCCGCA-3′, and 5′-CACAACGGGTTCTTCTGTTAGTCC-3′ for *Crisp4*. The expected size of the PCR product is ~894 bp for the wild-type (WT) allele and ~611 bp for the null allele for *Crisp1* and ~470 bp for the WT wild-type allele and ~650 bp for the null allele for *Crisp4*. All animals used in experiments were genotypified to ensure mutations in *Crisp1* and *Crisp4* genes.

### Assessment of fertility

WT, CRISP4 KO or DKO males (2 to 6 months old) were individually caged with one WT or DKO female for 4 days and mating was confirmed by the presence of copulatory plug. Each male was bred with at least 2 females and their fertility was calculated as average litter size.

### Assessment of estrous cycle and hormone treatment

Estrous cycle stage of sexually mature females was determined every morning by the analysis of vaginal smears^81^. Briefly, vaginal epithelial cell smears were collected in 30 ml of phosphate-buffered saline with a pipette, transferred to glass slides and examined unstained under a light microscope (×100). The different stages (pro-estrous, estrous, metaestrous and diestrous) were classified based on the proportion of the three cell types, i.e. leukocytes and both cornified and nucleated epithelial cells. For ovulation induction (*in vitro* studies), adult females were treated with an i.p. injection of equine chorionic gonadotrophin (eCG; 5 IU; Syntex SA, Buenos Aires, Argentina) at any stage of the cycle followed by an i.p. injection of human chorionic gonadotrophin (hCG; 5 IU, Sigma) 48 h later.

### *In vivo* fertilization assays

Sexually mature (2 to 6 months old) WT, CRISP4 KO or DKO males were individually caged for 18 h with one natural estrous female. Each male was subjected to two rounds of mating. Females showing presence of copulatory plug were then sacrificed and eggs were recovered from the oviducts, fixed with 2% paraformaldehyde, washed, stained with 10 µg/ml Hoechst 33342 (Sigma), mounted on slides and, finally, analyzed under a Nikon Optiphot microscope (Nikon, Tokyo, Japan) equipped with epifluorescence optics (×200). Eggs were considered fertilized when at least one decondensing sperm nucleus or two pronuclei were observed in the egg cytoplasm.

### Sperm collection and *in vitro* capacitation

Sperm were recovered from young adult males by incising cauda epididymides in 300 μl of capacitation medium^82^ supplemented with 0.3% of bovine serum albumin (BSA) under paraffin oil. In all cases where sperm parameters were analyzed, the cells were recovered from the two cauda epididymides of the animal to better mimic the *in vivo* condition where the ejaculate contains sperm coming from the two epididymides. After 10 min of incubation, sperm were counted and aliquots of the suspension (fresh sperm) were added to 300 μl of fresh medium previously placed in tissue culture dishes to give a final concentration of 0.1–1 × 10^7^ cells/ml. Sperm suspensions were then incubated for 90 min under paraffin oil at 37 °C in an atmosphere of 5% CO2 in air.

### Evaluation of sperm functional parameters

#### Sperm motility

10 μl of fresh sperm suspensions were placed on prewarmed slides and sperm movement was recorded by video microscopy. The percentage of motile sperm was calculated by analyzing a minimum of three hundred cells distributed in at least twenty different microscope fields.

#### Viability assessment

10 μl of fresh sperm suspensions were placed on pre-warmed slides with 10 ul of pre-warmed eosin and analyzed under a light microscope (×400).

#### Spontaneous and progesterone-induced acrosome reaction

The acrosomal status of capacitated sperm was evaluated by Coomassie Brilliant Blue staining as previously described^83^. Four hundred spermatozoa were evaluated in each treatment under a light microscope (×400). Sperm were scored as acrosome-intact when a bright blue staining was observed in the dorsal region of the head or as acrosome-reacted when no labeling was observed. For induction of the acrosome reaction, sperm were exposed to progesterone (30 μM final concentration; Sigma) in dimethylsulfoxide (DMSO) for the last 15 min of incubation. As a control, capacitated sperm were exposed only to DMSO (spontaneous acrosome reaction).

### Computer-assisted sperm analysis (CASA)

Sperm aliquots (15 μl) were placed between pre-warmed slides and cover slips (22 × 22 mm) to create a chamber with ~30 µm depth and were examined at 37 °C using the ISAS® (Integrated Semen Analysis System) v1.2 CASA system (Proiser R&D, S.L., Valencia, Spain). For each sample, a minimum of two hundred cells distributed in at least twenty different microscope fields were scored (30 frames acquired at 60 Hz for each measurement). Sperm were considered hyperactivated when presenting curvilinear velocity (VCL, µm/s) ≥ 238.5, linearity (LIN, %) < 33% and amplitude of lateral head displacement (ALH, µm) ≥ 4.22. These custom cutoffs were selected for our experimental conditions based on previously reported recommendations^84^.

### *In vitro* fertilization assays

#### Recovery and treatment of oocytes

Control female mice (2–6 month of age) were superovulated as indicated above. Oocytes were collected from the oviducts of superovulated animals 12–13 h after hCG administration. Cumulus cells were removed by incubating the oocyte–cumulus complexes for 3 min in 0.3 mg/ml hyaluronidase (type IV; Sigma). The ZP was dissolved by treating the oocytes with acid Tyrode solution (pH 2.5) for 10–20 s^85^.

#### Gamete co-incubation

Intact cumulus oocyte complexes (COC) or cumulus free ZP-intact eggs were inseminated with capacitated sperm (final concentration 0.5–2 × 10^5^ sperm/ml) and gametes were co-incubated for 3h at 37 °C in an atmosphere of 5% CO2 in air. ZP-free eggs were inseminated with capacitated sperm (final concentration: 0.5–1 × 10^4^ sperm/ml) and gametes co-incubated for 1 h. In all cases, eggs were then washed, fixed with 2% paraformaldehyde, and stained with 1 μg/μl Hoechst 33342 (Sigma). Finally, eggs were mounted on slides and analyzed under a UV microscope (×200). Eggs were considered fertilized if two pronuclei or decondensing sperm nucleus were observed in the ooplasm. In some cases, co-incubation of COC with sperm was allowed to continue for 24 h to obtain 2-cell embryos.

### Cumulus penetration assay

COC were inseminated with capacitated sperm (final concentration: 1–2.5 × 10^4^ sperm/ml) previously stained with 3 µg/ml Hoechst 33342 and gametes were co-incubated for 15 min at 37 °C in an atmosphere of 5% (v/v) CO2 in air. COC were washed and fixed as described above and the number of sperm within the cumulus was determined under the Nikon Optiphot microscope equipped with epifluorescence optics (×200).

### Sperm-ZP binding assay

ZP-intact eggs and 2-cell embryos (used as control) were co-incubated with capacitated sperm (final concentration: 1 × 10^5^ cells/ml) for 30 min at 37 °C in an atmosphere of 5% (v/v) CO_2_ in air. Eggs and embryos were washed thoroughly until no sperm remained bound to the embryos. Finally, eggs were fixed and the number of sperm bound to the ZP was counted under a light microscope (×400).

### Immunoblot analysis

Epididymides were homogenized in 1.5 volumes of ice-cold 50 mM Tris-HCl buffer (pH 7.4) containing 0.2 mM phenylmethylsulphonyl fluoride (PMSF). The homogenates were then centrifuged twice at 10,000 × g for 20 min at 4 °C and the supernatants were dialyzed against 50 mM Tris-HCl buffer (pH 6.8). Sperm aliquots (1 × 10^6^ spermatozoa) obtained before or after capacitation were washed with phosphate buffered saline (PBS) and resuspended in Laemmli sample buffer^86^. In all cases, after a 5 min-incubation, samples were boiled, centrifuged at 5000 × g for 5 min and the supernatants were recovered. For tyrosine phosphorylation assessment, the supernatants were boiled again in the presence of 70 mM 2-β-mercaptoethanol (Sigma).

Protein samples (50 μg/lane or 2 × 10^6^ sperm/lane) were separated by SDS–PAGE and transferred onto nitrocellulose membranes. After blocking with 2% skim milk in PBS, the membranes were probed with either polyclonal anti-mouse IDO (1:1000; Santa Cruz sc-25809), polyclonal anti-mouse Galectin-1 (1:750), anti-actin monoclonal antibody (1:2000; Santa Cruz sc-8432), anti-mouse CRISP4 (1:1000; R&D Systems #AF5017) polyclonal antibody, or anti-β tubulin monoclonal antibody (1:4000; clone D66, Sigma, St. Louis, MO, USA) followed by the corresponding peroxidase-conjugated secondary antibody (1:4000). The immunoreactive proteins were detected by an ECL Western Blotting kit (GE Healthcare UK Ltd, Buckinghamshire, England) and images captured with G:BOX GENI (Syngene, Synoptics Ltd, Cambridge, England) according to the manufacturer’s instructions.

### Histological examination of testes and epididymides

Testes and epididymides from adult males were removed, weighted and fixed for at least 24 h by immersion in Bouin solution. Tissues were then processed for paraffin embedding and sectioning by routine methods. Sections were stained with hematoxylin-eosin solutions and examined by light microscopy (×100 or ×400).

### Immunoperoxidase

Cryostat sections (6 mM thick) were fixed in cold paraformaldehyde 4% in PBS. Immunoperoxidase staining was performed using the avidin-biotin system (ABC Vectastain Kit, Vector Lab., Burlingame, CA, USA) to detect F4/80 antigen. Sections were washed in PBS and incubated with 5% skim milk, 0.01% Triton X-100, and then treated with avidin–biotin blocking solution (Vector Laboratories). After overnight incubation with a rat anti-mouse F4/80 antibody (1:5) (Tonbo Biosciences, San Diego, CA, USA) at 4 °C in a humidified chamber, sections were incubated with biotinylated anti-rat IgG (1:250, Vector Lab.). Endogenous peroxidase activity was blocked by treatment with 0.3% H_2_O_2_ in methanol for 30 min. Then, the reaction was amplified using ABC Vectastain kit and the 3-3′diaminobenzidine-H_2_O_2_ (DAB Substrate Kit, Vector Lab.) was used as peroxidase substrate. Sections were counterstained with hematoxylin.

### Immunofluorescence

Epididymides were removed and fixed by immersion in paraformaldehyde 4% in PBS for 3 h at room temperature, washed in PBS, and stored at 4 °C in PBS containing 0.02% sodium azide. Fixed tissues were cryoprotected in PBS with 30% sucrose for at least 24 h at room temperature, embedded in Tissue-Tek OCT compound (Sakura Finetek, Torrance, CA), and mounted and frozen on a cutting block. Tissues were cut in a Reichert Frigocut microtome at 7 μm thickness and sections were placed onto Fisherbrand Superfrost Plus microscope slides (Fisher Scientific, Pittsburgh, PA), processed as previously described^87^ and incubated with different antibodies: anti-AQP9^50^ (1:200), anti-V-ATPase^53^ (1:800), anti-KRT5 (Abcam, ab53121; 1:500) The corresponding secondary antibodies were donkey anti-chicken IgG conjugated to Alexa488 1:100, donkey anti-rat IgG conjugated to FITC 1:100 and donkey anti-rabbit IgG conjugated to indocarbocyanine (Cy3) 1:800 (Jackson Immunologicals, West Grove, PA).

### Quantitative Reverse Transcriptase Polymerase Chain Reactions (qRT-PCRs)

Total RNAs from mouse epididymis were isolated with Trizol (Gibco) according to the manufacturer’s recommendations and were reverse transcribed by M-MLV Reverse Transcriptase (Promega Corp., France) according to the manufacturer’s instructions. RT was performed using 2 μg of total RNA, and the resulting cDNA was used for qPCR analysis which was performed using specific primers and the FastStart Universal SYBR Green Master (Sigma-Aldrich) on an iCycler Thermal Cycler (Bio-Rad). Target gene expression was quantified by comparing the threshold cycle (CT) with that of cyclophilin by using the comparative CT method (ΔΔCT). The primer sequences used for qPCR were complementary to different exons of the following mouse genes:

*Ido*: Fwd: 5′-ACTGTGTCCTGGCAAACTGGAAG-3′; Rvs: 5′-AAGCTGCGATTTCCACCAATAGAG-3′

*Cyclophilin*: Fwd: 5′-GACCCTCCGTGGCCAACGAT-3′; Rvs: 5′-ACGACTCGTCCTACAGATTCATCTC-3′

*Il-6*: Fwd: 5′-CCTCTCTGCAAGAGACTTCCA-3′; Rvs: 5′-ACAGGTCTGTTGGGAGTGGT-3′

*Il-10*: Fwd: 5′-TTCCCTGGGTGAGAAGCTGA-3′; Rvs: 5′-CTTCACCTGCTCCACTGCCT-3′

*TGF-β*: Fwd: 5′-AATTCCTGGCGTTACCTTGG-3′; Rvs: 5′-ATCGAAAGCCCTGTATTCCG-3′

### Measurements of pH in epididymal fluids

Epididymal pH was measured by using pH strips as this method provides as good results as those obtained using electrodes with the benefit that it allows to measure the pH immediately after release of fluids from the epididymal lumen avoiding any loss of carbon dioxide in the time during release, accumulation for collection, and measurement^55^. Briefly, different epididymal regions (caput, corpus and cauda) were isolated and lobules of tubules were dissected free of the capsule to avoid blood vessels. Tubules were cut at a few sites and gently pressed so that the exuded luminal contents were immediately smeared onto a pH strip and read (with 0.3-unit increments in the reference colors, strips from Sigma).

### Calculations and statistical analysis

Data represent the mean ± SEM from at least three independent experiments. Calculations were performed using the Prism 4.0 software (GraphPad Software, La Jolla, CA, USA). *In vitro* fertilization, cumulus penetration, ZP-binding and hyperactivation were analyzed by the t-student test. In all other cases, comparisons were evaluated by two-way ANOVA. Differences were considered significant at a level of P < 0.05.

## Electronic supplementary material

Supplementary file

Full length Blots
