# Supplementary material for: Impaired male fertility and abnormal epididymal epithelium differentiation in mice lacking CRISP1 and CRISP4
Source: Sci Rep. 2018 Dec 3;8:17531. doi: 10.1038/s41598-018-35719-3 (PMC6277452; doi:10.1038/s41598-018-35719-3)
Supplement: Supplementary file 1 — Supplementary file [file 41598_2018_35719_MOESM1_ESM.docx]

**Impaired male fertility and abnormal epididymal epithelium differentiation in mice lacking CRISP1 and CRISP4**

Carvajal Guillermo, Brukman, Nicolás G, Weigel Muñoz Mariana, Battistone María A, Guazzone Vanesa A, Ikawa Masahito, Haruhiko Miyata, Lustig Livia, Breton Sylvie, Cuasnicu Patricia S

**Supplementary information**


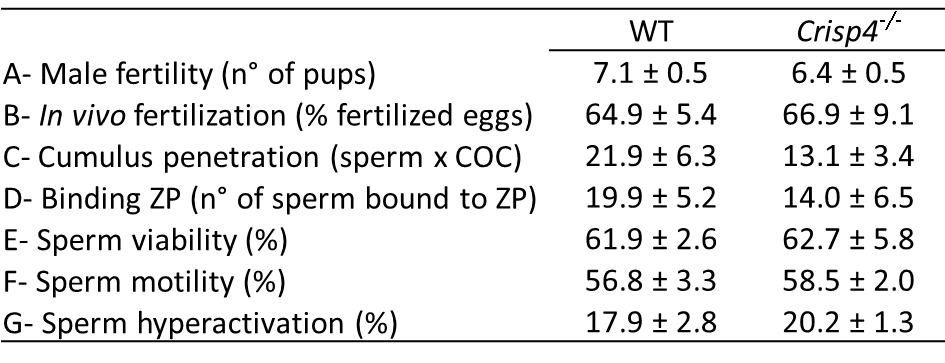


**Table S1. *Crisp4^-/-^* phenotype in C57BL/6*DBA background: A)** WT or *Crisp4 ^-/-^* adult males were bred with control females for 4 days and the number of pups was analyzed. Data are mean ± SEM, *n=9, ns.* **B)** WT and *Crisp4 ^-/-^* males were mated with hormone stimulated females and the percentage of fertilized eggs recovered from the ampulla was evaluated the following day. Data are mean ± SEM, *n=5, ns.* **C)** Hoescht-stained capacitated sperm were co-incubated with COC for 15 min and the number of sperm within the cumulus matrix was determined. Data are mean ± SEM, *n=5, ns.* **D)** Capacitated sperm were co-incubated with ZP-intact eggs for 30 min and the number of sperm bound to the ZP was evaluated. Data are mean ± SEM, *n=5, ns*. **E)** Cauda epididymal sperm were labeled with eosin and their viability analyzed under a light microscope (x400). Data are mean ± SEM, *n=6, ns.* **F)** Analysis of progressive motility of cauda epididymal sperm by CASA. Data are mean ± SEM, *n=5, ns*. **G)** Capacitated cauda epididymal sperm were evaluated by CASA and hyperactivation levels were determined. Data are mean ± SEM, *n=5, ns*.


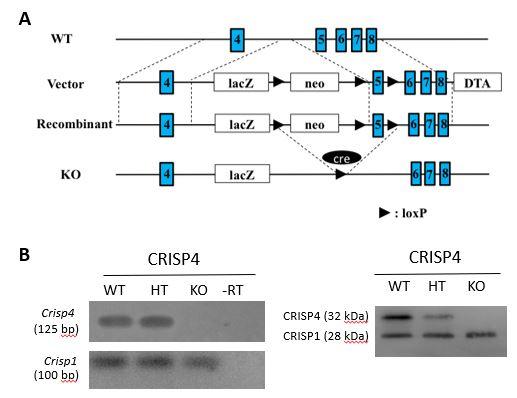


**Figure S1. Disruption of the mouse *Crisp4* locus.** **A:** Schematic representation of the wild type allele, targeting vector, and both recombined and KO alleles. The blue rectangles and numbers within them denote the exons in the *Crisp4* gene. The open boxes represent the negative (lacZ) and positive (neo) selection cassettes. To remove the floxed-exons, F1 mice were mated with B6D2 CAG-Cre transgenic animals that ubiquitously express Cre. lacZ: beta galactosidase. neo: neomycin resistance cassette. DTA: diphtheria toxin A chain. **B:** RT-PCR (left) and western blotting (right) images showing the lack of expression of CRISP4 in the epididymis of the mutant mice. WT: wildtype, HT: heterozygous, KO: knockout. -RT: without retrotranscriptase


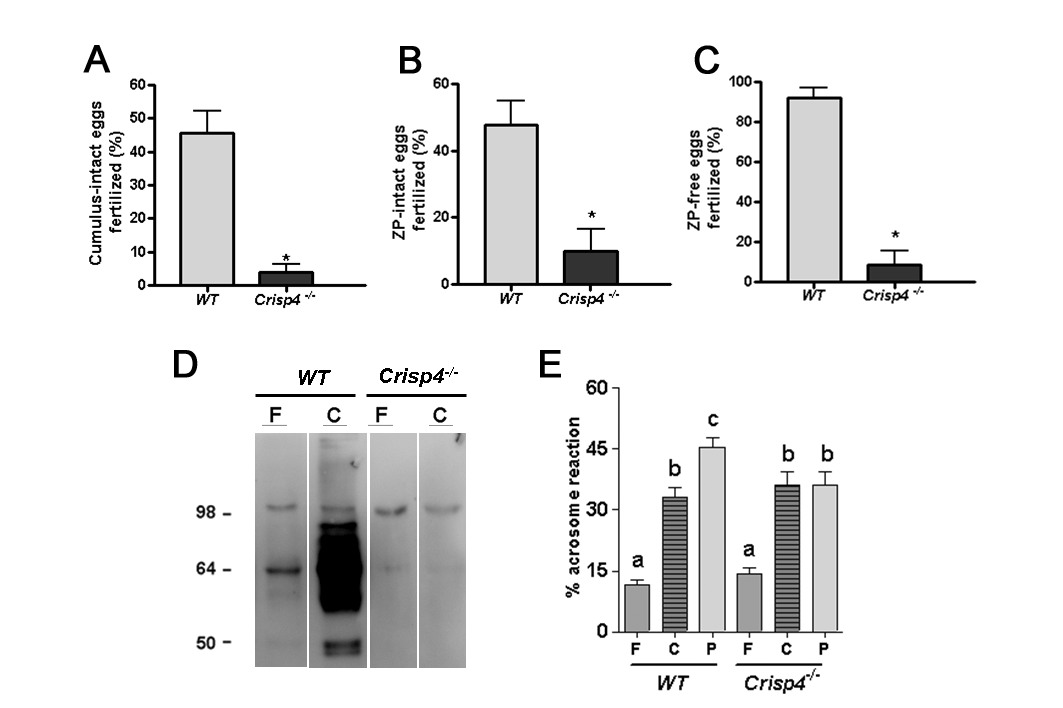


**Figure S2. *Crisp4^-/-^* phenotype in C57BL/6*DBA background:** WT and *Crisp4 ^-/-^* sperm were incubated under capacitating conditions for 90 min and different functional parameters evaluated. Sperm were co-incubated with **(A)** COC for 3 h, **(B)** ZP-intact eggs for 3 h, or **(C)** ZP-free eggs for 1 h. At the end of all incubations, fertilization was evaluated by the presence of decondensing sperm heads within the egg cytoplasm. Data are mean ± SEM*, n=5, p*<0,001*. **D)** Protein tyrosine phosphorylation was analyzed by western blotting using an anti-phosphotyrosine antibody. A representative blot is shown*, n=4*. **E)** Percentage of acrosome reaction determined by Coomassie Brilliant Blue staining in fresh (F) and capacitated sperm exposed to either progesterone (P) or dimethyl sulfoxide alone (C) during the last 15 min of incubation. Data are mean ± SEM, *n=10;* Different letters indicate significant differences between groups (a vs b, *p<0.01;* a vs c, *p<0.001;* b vs c, *p<0.05*).


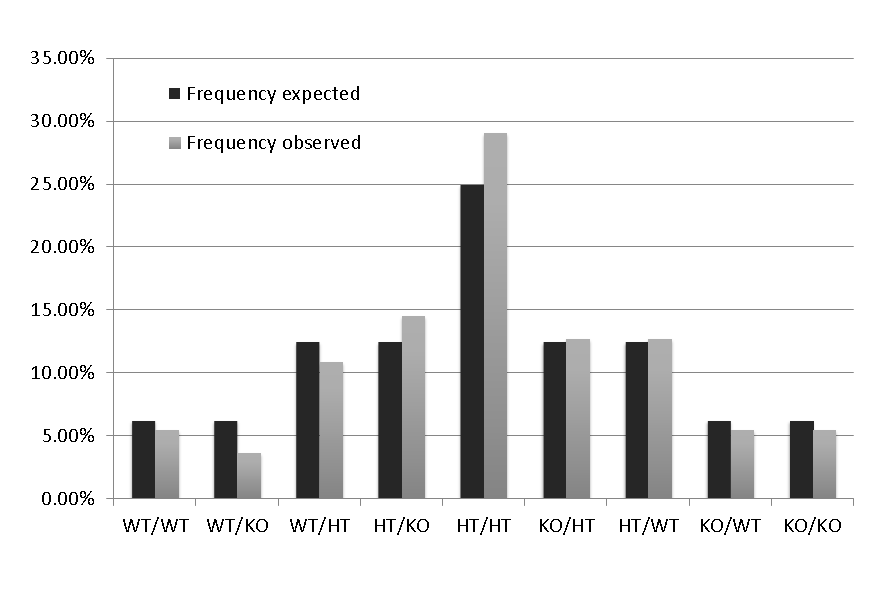


**Figure S3. Genotype distribution of DKO mice:** Pups born from mating of HT/HT males with HT/HT females were genotyped. Analysis of the amount of mice of each genotype revealed the expected Mendelian distribution.


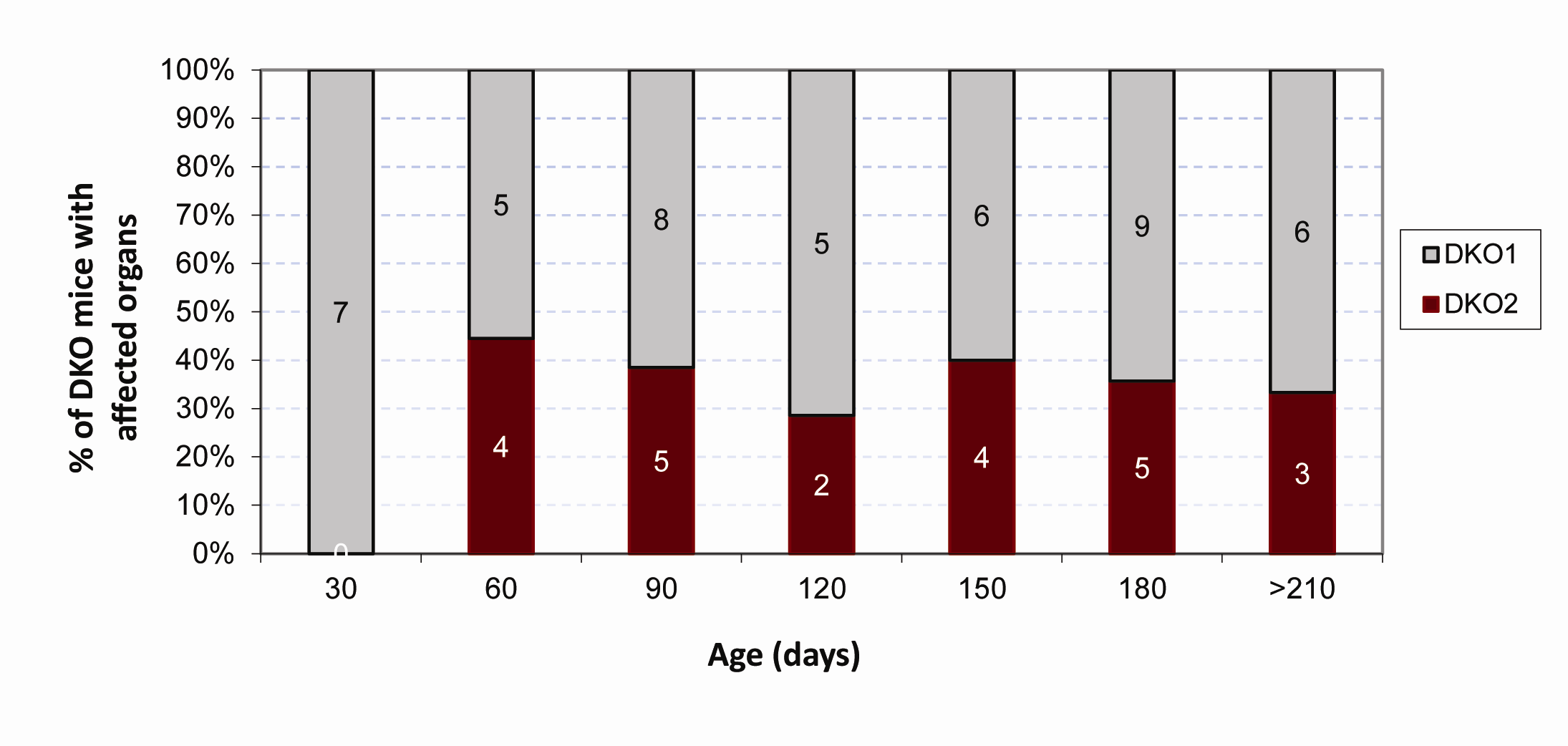


**Figure S4. Effect of age on the incidence of males with bigger testes and epididymides:** Percentage of animals exhibiting affected (DKO2) or non-affected (DKO1) reproductive organs as a function of animal age. The numbers inside the bars indicate the amount of males exhibiting each phenotype.


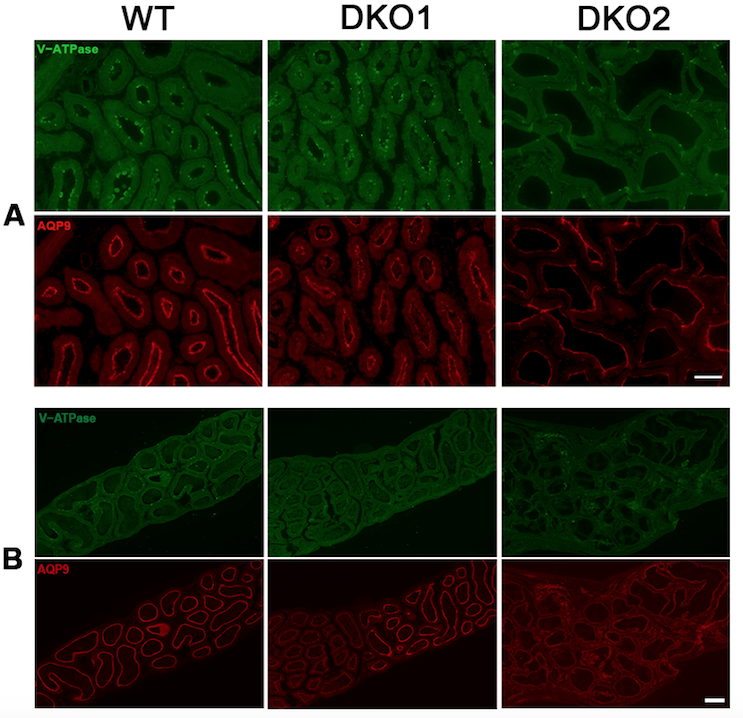


**Figure S5. Effect of the lack of CRISP1 and CRISP4 on epididymal epithelium phenotype.** Double immunolabeling of AQP9, marker of principal cells (red) and V-ATPase B1 subunit, marker of clear cells (green) in the caput (A) and corpus (B) epididymis of WT and of both Group1 (DKO1) and Group 2 (DKO2) mutant males. Representative images are shown. Scales correspond to 100 (A) and 200 µm (B).
