# Supplementary material for: Impaired male fertility and abnormal epididymal epithelium differentiation in mice lacking CRISP1 and CRISP4
Source: Sci Rep. 2018 Dec 3;8:17531. doi: 10.1038/s41598-018-35719-3 (PMC6277452; doi:10.1038/s41598-018-35719-3)
Supplement: Supplementary file 2 — Full length Blots [file 41598_2018_35719_MOESM2_ESM.pdf]

Impaired male fertility and abnormal epididymal epithelium differentiation in mice lacking CRISP1 and CRISP4

Carvajal Guillermo, Brukman, Nicolás G, Weigel Muñoz Mariana, Battistone María A, Guazzone Vanesa A, Ikawa Masahito, Haruhiko Miyata, Lustig Livia, Breton Sylvie, Cuasnicu Patricia S

Supplementary file

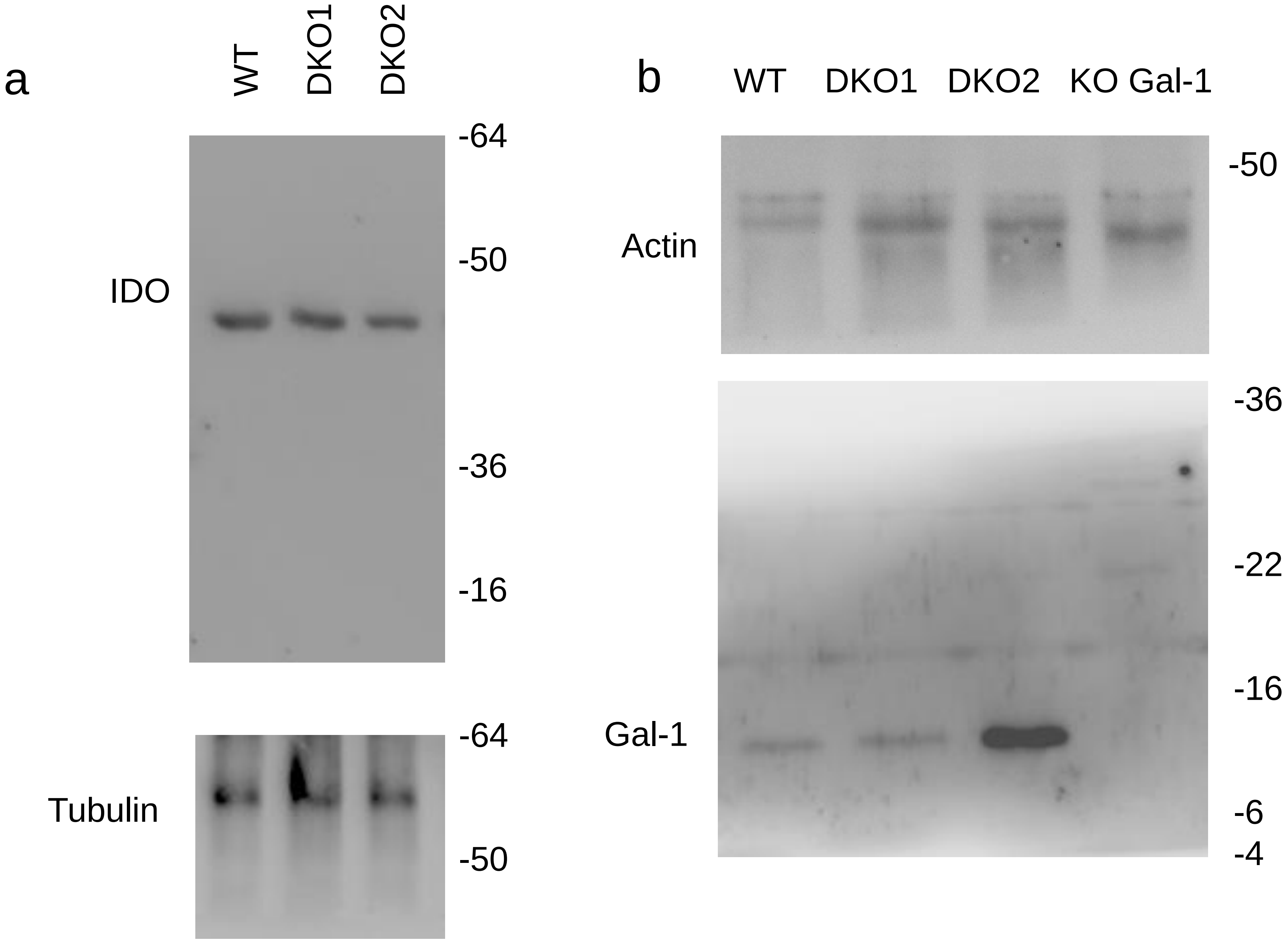
a
WT
DKO1
DKO2
-64
-50
IDO
-36
-16
-64
Tubulin
-50
b
WT
DKO1
DKO2
KO Gal-1
-50
Actin
-36
-22
-16
Gal-1
-6
-4
